# Symbolic flux analysis for genome-scale metabolic networks

**DOI:** 10.1186/1752-0509-5-81

**Published:** 2011-05-23

**Authors:** David W Schryer, Marko Vendelin, Pearu Peterson

**Affiliations:** 1 Laboratory of Systems Biology, Institute of Cybernetics at Tallinn University of Technology, Akadeemia tee 21, 12618 Tallinn, Estonia

## Abstract

**Background:**

With the advent of genomic technology, the size of metabolic networks that are subject to analysis is growing. A common task when analyzing metabolic networks is to find all possible steady state regimes. There are several technical issues that have to be addressed when analyzing large metabolic networks including accumulation of numerical errors and presentation of the solution to the researcher. One way to resolve those technical issues is to analyze the network using symbolic methods. The aim of this paper is to develop a routine that symbolically finds the steady state solutions of large metabolic networks.

**Results:**

A symbolic Gauss-Jordan elimination routine was developed for analyzing large metabolic networks. This routine was tested by finding the steady state solutions for a number of curated stoichiometric matrices with the largest having about 4000 reactions. The routine was able to find the solution with a computational time similar to the time used by a numerical singular value decomposition routine. As an advantage of symbolic solution, a set of independent fluxes can be suggested by the researcher leading to the formation of a desired flux basis describing the steady state solution of the network. These independent fluxes can be constrained using experimental data. We demonstrate the application of constraints by calculating a flux distribution for the central metabolic and amino acid biosynthesis pathways of yeast.

**Conclusions:**

We were able to find symbolic solutions for the steady state flux distribution of large metabolic networks. The ability to choose a flux basis was found to be useful in the constraint process and provides a strong argument for using symbolic Gauss-Jordan elimination in place of singular value decomposition.

## Background

The explosion of tools available to simulate the systems level properties of biological systems is indicative of the wide scale uptake of integrative biology. The Systems Biology Markup Language (SBML) Web site [1] now lists over 200 packages that make use of their library. This large number of tools reflects both the wide variety and abundance of biological data now available to constrain biological models as well as the large variety of simplifying assumptions made to gain insight from this plethora of data.

At the core of many of these analytical tools is the strict requirement of conservation of mass for each biological transformation. Because models of metabolic systems are typically under-determined, a common task when analyzing them is to find all possible steady state regimes when the concentrations of each metabolite do not change appreciably with time.

With the advent of genomic technology, the size of networks that are subject to conservation analysis is growing. This is true also of the amount of data that constrains biological function, forcing the analysis procedure to become more involved. This is especially true when faced with the realities of compartmentation in large biological systems.

The analysis of systems of chemical reactions can be traced back to 1921 when Jouguet established the notion of independence of reactions and the invariants of a system of reactions [2]. In the 1960s, with the advent of computers, routines were written for solving systems of chemical equations [3]. These were made accessible to biologists and opened up the possibility for simulating complex biological systems [4].

It may come as a surprise to many biologists that the mathematically simple operation of finding a set of parameters that describe the steady state solution of large chemical systems continues to challenge the limits of widely used numerical libraries used to perform this task, and the development of robust computational routines for this purpose continues to be an active research area [5]. Sauro and Ingalls reviewed a number of technical issues related to the analysis of large biochemical networks and mention the attractiveness of using rational arithmetic routines that avoid the accumulation of errors [6]. They point out that this symbolic approach requires a complete rewrite of the algorithms used to solve these systems. Programs that perform conservation analysis exist. A review [6] discusses 13 software packages that perform stoichiometric conservation analysis. However, only one of these (emPath by John Woods) uses rational arithmetic. For analyzing large metabolic networks the use of numerical algorithms with floating point arithmetics seems to be considered the only practical approach, especially because of the numerical robustness of singular value decomposition (SVD) algorithm that is an integral part of many analysis tools. A more recent study uses a Computer Algebra System for symbolic Metabolic Control Analysis [7]. The author notes that the most pertinent issue with symbolic computation is its inefficiency and for the analysis of very large systems more efficient methods and software need to be developed. Other methods exists to avoid floating point operations, for example, de Figueiredo et al use a linear integer programming approach to find the shortest elementary flux modes in genome scale networks [8]. Linear programming was also used to avoid exhaustive identification of elementary flux modes as well as problems in computing null-space matrices for large metabolic networks [9].

It is notable that existing software packages do not take into account the inherit sparsity of large metabolic networks [6]. This is most likely because the result of SVD is generally non-sparse and further analysis would require non-sparse data structures. So, the use of SVD based algorithms for large metabolic networks will be limited by the size of available computer memory. For example, creating a dense stoichiometric matrix with 4000 reactions takes approximately 100MB of computer memory and various matrix operations may increase the actual memory need by a factor of ten. Holding the same stoichiometric matrix in a sparse data structure is almost one thousand times more memory efficient (Recon 1 [10] has a sparsity of 99%, for instance).

To our knowledge, no software package is available that both makes use of rational arithmetic and accounts for the inherit sparsity of large metabolic networks. To use sparse representations of metabolic networks, SVD based algorithms need to be replaced with alternative algorithms that would preserve the sparsity property in their results. To achieve the same numerical robustness of these algorithms as SVD provides, rational arithmetics can be used. The decrease of performance due to the use of rational arithmetics ought to be balanced by the sparsity of matrices as the number of numerical operations is reduced considerably. The aim of this paper is to develop a routine that symbolically finds the steady state solutions of large chemical systems.

Specifically, we have developed a routine that solves for the kernel of large stoichiometric matrices using a symbolic Gauss-Jordan Elimination (GJE) routine. For a given metabolic network the routine computes steady state solutions in a form of steady state flux relations that define how certain fluxes termed as dependent fluxes vary when the rest of fluxes termed as independent fluxes are changed. The list of dependent and independent flux variables can be either computed by the routine or specified by the researcher. The performance of this method is compared with Singular Value Decomposition (SVD) implemented in a widely used numerical routine. In addition, we demonstrate that the usefulness of solving for the stoichiometric matrix kernel symbolically goes beyond the avoidance of numerical errors. Specifically, the kernel arrived at using GJE consists of flux vectors that align with actual metabolic processes which is useful for applying constraints on steady state metabolism.

## Results

A symbolic GJE routine was developed within SympyCore [11] during the course of this research. This routine was tested by finding the kernels for a number of curated metabolic models, and then utilized to calculate a metabolic flux distribution for the central metabolic and amino acid biosynthesis pathways of yeast.

### Comparison of GJE and SVD

Five large metabolic networks of increasing complexity were selected to test the performance of symbolic GJE to that of numerical SVD. These metabolic networks were formulated in a closed form as described by Famili and Palsson [12]. To obtain non-trivial solutions to the steady state equations, the metabolic networks need to be converted to open form where the boundary conditions are specified via transport fluxes into the network rather than via external metabolites. For simplicity, we convert the metabolic networks to open form by introducing transport fluxes across the network boundary to metabolites that either appear in exactly one reaction or are products of polymerization reactions (see Methods).

**Figure 1:**
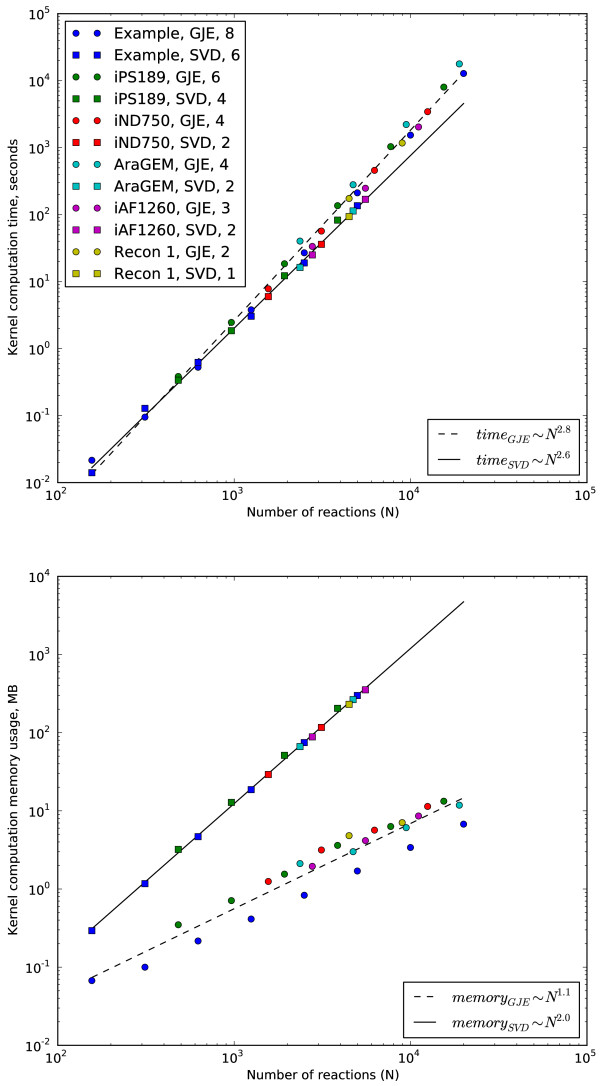
**Computational resources for computing kernels**. The computational time (upper) and memory usage (lower) for computing kernels of stoichiometric matrices using SVD and GJE algorithms for curated genome-scale networks. The system names correspond to those from Table 1. The squares correspond to SVD while circles to GJE. Numbers in upper legend denote the number of duplicated versions of the same network (see Results). Note that the computational time increases with increasing network size and the growth rate is roughly the same for both methods. However, SVD memory usage increases at twice the rate of GJE memory usage.

The kernel of five stoichiometric matrices were solved for using both numerical SVD and the symbolic GJE routine with the results given in Table 1. The computation time for both methods was found to be almost the same with SVD being slightly faster. However, we noted that the numerical SVD routine used effectively two CPUs (see Methods for details about the test computer system) while the symbolic GJE routine used only one. Hence for a number of parallel kernel calculations that would consume all computer CPUs, the symbolic GJE routine would be more productive. Figure 1 (top) illustrates how the kernel computation time depends on the size of the network. The computational time increases exponentially with the size. It should be noted that the ratio of these exponents depends on a computer system and underlying computational libraries. Also note that the complexity of both SVD and GJE algorithms are *O*(*mn*^2^), that is, increasing the network size by a factor of 10, the complexity should increase by 1000 times. The actual complexity increase (about 400 for SVD and 640 for GJE) is smaller because of using threaded libraries for the SVD routine and because of computing with high sparsity for the GJE routine. The numerical errors introduced when using SVD were found to be insignificant for the purpose of biological flux calculations and confirm the fact of numerical robustness of the SVD routine. This assessment was made by calculating the maximum relative flux error *ε*_SVD _using Equation (11). Note that this loss of accuracy is in agreement with the condition number calculated for **V**_indep _in Equation (9); the number of inaccurate digits is approximately equal to the order of magnitude of *ε*_SVD_.

**Table 1:** Performance of GJE versus SVD

Model	**Publ**.	Species	Reactions		Flux variables		CPU time (s)		*ε*SVD ×10^-12^	Condition number
			**Orig**.	Open	**Dep**.	**Indep**.	SVD	GJE		
Example		118	129	156	118	39	0.02	0.03	0.003	15
iPS189	[15]	433	350	482	413	69	0.3	0.4	0.07	31000
iND750	[16]	1177	1266	1561	1162	399	6.2	8.0	6.37	68000
AraGEM	[17]	1767	1625	2361	1720	641	19.7	34.2	12.65	3000
iAF1260	[18]	1972	2382	2773	1960	813	30.5	34.3	1.43	2800
Recon 1	[10]	3188	3742	4480	3169	1311	123.5	145.6	32.63	71000

Kernel computation times for numerical SVD and symbolic GJE for the example yeast network given in Figure 3 and five genome-scale metabolic networks. All techniques are described in Methods. The condition number was calculated for **V**_indep _from Equation (9). The inversion of **V**_indep _is required to directly compare SVD results with the solution found from GJE. The difference between the results is given by *ε *_SVD _in Equation (11).

**Figure 2:**
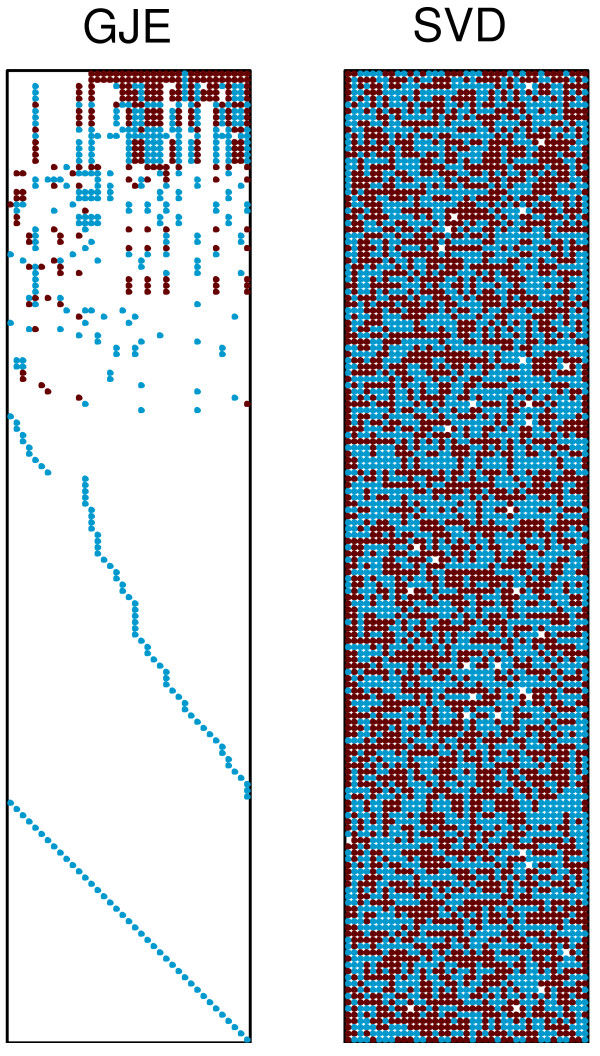
**Kernels for the example yeast network**. Two kernels of the stoichiometric matrix of the example yeast network obtained with SVD (left) and GJE (right) algorithms, respectively. The kernels define the same steady state solutions but the sparsity of the GJE kernel allows easier interpretation of these solutions.

With our test computer system both numerical SVD and symbolic GJE routines can easily cope with 4000+ reaction networks. To test the limits of these routines, we repeatedly doubled the sizes of considered networks by repeating given stoichiometric matrix diagonally within a doubled stoichiometric matrix and then randomly shuffling the columns. The doubled stoichiometric matrix would then correspond to two independent but identical metabolic networks. The shuffling is needed for modeling the structure of actual metabolic network models where the order of columns is arbitrary. The process of increasing the sizes of networks was repeated with doubled stoichiometric matrices until applying our routines were close to exceeding the resources of our computer system. Figure 1 (bottom) shows the dependence of the memory usage on the size of the network. The memory usage for computing the kernels increases exponentially with the size. The two times smaller memory increase when using the symbolic GJE routine compared to the numerical SVD routine is explained by the fact that symbolic GJE routine preserves sparsity while the result of numerical SVD routine is generally non-sparse. This is illustrated in Figure 2 where the corresponding kernels from SVD and GJE algorithms are shown for the example yeast network (see next Section). For other tested networks the sparsity of GJE kernels varied in the range 95-99.9% and the sparsity of SVD kernels in 1-25%.

### Application of constraints to the example yeast network

**Figure 3:**
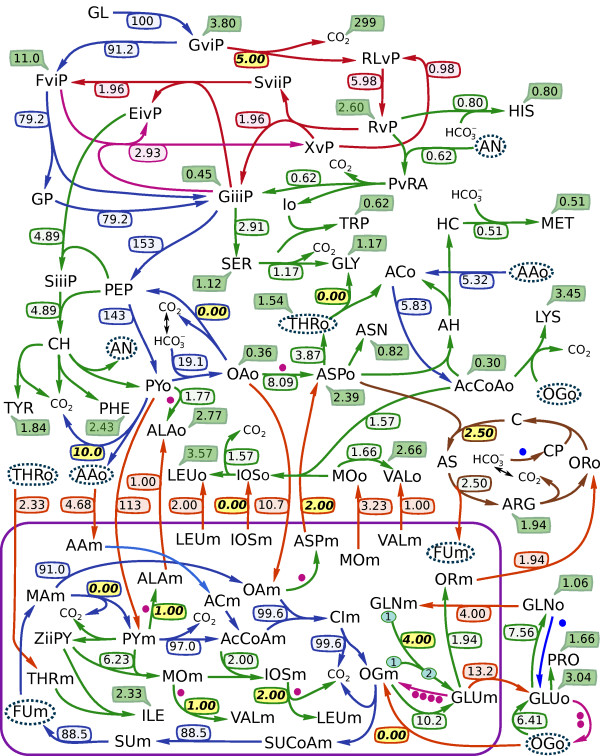
**Example yeast network**. One flux distribution for the central metabolic and amino acid biosynthesis pathways of yeast. Metabolite abbreviations, reaction details, and the symbolic flux relations used to calculate this steady state are provided in additional file 2: yeast_example.pdf. The values of the independent flux variables substituted into the flux relations are set in italic font. The mitochondrial compartment is separated with a purple boarder and all inter-compartmental transport reactions are given as orange arrows. Amino acid synthesis reactions are green, and all transport fluxes out of the system are depicted with green cartoon bubbles. The pentose phosphate pathway reactions are given in red and the urea cycle is shown in brown. Dots are placed next to reactions that are coupled; pink dots indicate the transformation of glutamate to oxoglutarate, and the blue dot shows the transformation of glutamine to glutamate. Species that occur in more than one place within one compartment are circled with a dotted blue line.

Often one needs to constrain the flux values that are physiologically meaningful, that is, either they have been experimentally measured or they must be non-negative due to the irreversibility of some reactions. We demonstrate the application of constraints by calculating a flux distribution for an example yeast network. The metabolic network is given as an SBML file in additional file 1: yeast_example.xml, and is laid out in Figure 3. This network contains 129 reactions and 118 metabolites, including 62 metabolites in the cytosol, 29 metabolites in mitochondria, and 27 metabolites that are external to the network. Because the list of external metabolites is known in this example then the system can be converted to open form by removing those rows from the stoichiometric matrix that correspond to external metabolites. Note that this is our alternative method of opening metabolic networks (see Methods).

The symbolic GJE of the stoichiometric matrix for the open system provides 91 relations for the dependent fluxes expressed in terms of 39 independent fluxes. A full list of reactions, metabolites, and steady state flux relations is given in additional file 2: yeast_example.pdf. The corresponding kernel matrix is shown in Figure 2. The relations are formed from the rows of this matrix.

The independent fluxes can be selected prior to performing GJE. We compose the set of independent fluxes from biomass production rates that have been experimentally measured. In total, 26 measured biomass fluxes taken from Cortassa et al [13] were used to constrain this network. The table of such exchange fluxes and their values is given in additional file 2: yeast_example.pdf. The remaining 13 independent flux variables are left unspecified which means that the symbolic GJE routine will choose a viable set of independent fluxes. In this example these are all internal to the network.

After substituting the biomass production values into the steady state flux relations, 27 dependent fluxes become fully specified with 64 relations described by the 13 internal variables. Inspection of this system of equations (also given in additional file 2: yeast_example.pdf) immediately reveals which part of the metabolism each of these 13 variables controls. Each internal variable is connected to dependent variables via nonzero entries in the corresponding column of the kernel matrix. The set of these dependent variables share metabolites and thus can be considered as one connected sub-network of the original system. Five of these sub-networks determine the split between cytosolic and mitochondrial valine, leucine, alanine, and aspartate biosynthesis via *BAT1 *and *BAT2*, the split between *LEU4 *and *LEU9*, *ALT1 *and *ALT2*, and *AAT1 *and *AAT2*. Two determine the interconversion and transport of glutamine, glutamate, and oxoglutarate via the split between *GLT *and *GDH*. The remaining six determine: (1) urea cycle flux, (2) relative production of glycine from either serine or threonine, (3) the flux of D-Glucose 6-phosphate directed towards D-Ribulose 5-phosphate, (4) production of pyruvate by the malic enzyme *MAE*1, (5) the production of phosphoenolpyruvate by *PCK*1, and (6) the relative production of acetaldehyde to acetyl-CoA from pyruvate. Figure 3 gives one flux distribution calculated by specifying the values for the 26 biomass fluxes and 13 internal fluxes. The values chosen to substitute into the flux relations are highlighted on the figure.

In addition to constraining the measured independent variables directly, knowledge about the dependent fluxes in the example yeast network was used to constrain the network. We specified the net flux direction for reactions that involved the production of carbon dioxide. The constraints of measured flux values and the specified net flux direction of reactions, can be written as a system of 91 flux relations, 26 measured independent fluxes, and 17 inequalities. Following this all redundancies were removed using computational geometry techniques described in Methods. The result is a set of five upper and lower bound conditions for 5 independent fluxes, given in additional file 2: yeast_example.pdf.

## Discussion

It is now computationally practical to find the kernel of large stoichiometric matrices symbolically. The computational expense of symbolic GJE was not found to be overly restrictive with SympyCore [11], the package we used for analyzing genome-scale metabolic networks. The kernel obtained using the symbolic approach avoids numerical errors that may occur when applying numerical methods. The numerical errors result from the multitude of row operations that are needed to decompose large stoichiometric matrices [6]. The maximum relative flux error presented in Table 1 was found to be insignificant for biological flux calculations. However, symbolic GJE was found to be useful in more ways than avoiding numerical errors.

### Symbolic relationships give an informative representation of metabolic network structure

There are several technical issues that complicate analysis of large metabolic networks. Among them are numerical robustness of the algorithm and presentation of solution to the researcher [5,6]. Those problems are resolved when using symbolic GJE presented in this work. While GJE and SVD provide mathematically equivalent methods of solving for the steady state flux relations of metabolic networks, there is a difference in how the solutions are formed. In SVD, steady state solution is given through a combination of eigenvectors that often span the entire metabolic network [12]. Those eigenvectors contain information about the metabolic network, however extracting and interpreting this information is not always trivial and has inspired the creation of a diverse set of tools and techniques [14]. In contrast, symbolic GJE gives the researcher an opportunity to find the set of independent fluxes and relationships between independent and dependent fluxes. Through such relationships it is easy to see which dependent fluxes are influenced by any particular independent flux and gain insight into the operation of the metabolic network.

In the example yeast network given in Figure 3, many different sets of independent fluxes can be used to find a steady state solution. The GJE routine allows the researcher to specify which independent fluxes will be used to form the solution. By choosing biomass production rates, one can constrain the operation of the metabolic network to any given set of biomass measurements.

In our example, application of biomass constraints leaves 13 independent variables that are internal to the network and define all steady state flux distributions. We found that these 13 independent fluxes influence only a specific portion of the metabolism. Each independent variable only influences those dependent fluxes that have non-zero values in its column of the GJE kernel matrix. This property has potentially far reaching implications for the physical interpretation of steady state metabolism in large networks. All nonzero entries in each column of the GJE kernel define a set of dependent variables. These variables share metabolites and thus form a sub-network. Sub-networks that share common dependent variables can be combined into a larger sub-network. For example, it allows one to identify sub-networks within the metabolic network that are linked with shared metabolites and are controlled by sets of independent fluxes. In the example yeast network two fluxes are needed to describe glutamine, glutamate, and oxoglutarate transport and interconversion while five fluxes control the split between cytosolic and mitochondrial production of valine, leucine, alanine, and aspartate. The loops within these sub-networks are determined solely by independent fluxes that occur within each sub-network.

### Applicability of symbolic GJE and technical issues

We found that the computational time of applying symbolic GJE and numerical SVD routines to be similar for all networks considered. The memory usage of numerical SVD routine for networks with 6000+ reactions became close to exceeding memory resources of our test computer system. With the same memory usage level GJE routine would be able to analyze a network with 10^6 ^reactions, however, this calculation is estimated to take one year. Even when memory usage will be optimized in the SVD routine, the doubling network size will quadruple SVD memory usage while GJE memory usage would only double. This is because GJE algorithm preserves sparsity.

We did not observe the phenomena of coefficient explosion that would be typical for GJE algorithm using rational arithmetics on large matrices. This is explained because genome-scale stoichiometric matrices are inherently sparse and majority of elements are small integers such as 1 or -1. In addition, SympyCore [11] minimizes the number of operations by its pivot element selection rule (see Methods) to reduce computational time and this has added benefit of reducing the chance of coefficient explosion.

The reduced row echelon form of the stoichiometric matrix is formed by elementary row operations. The sequence of elementary row operations typically depends on the original ordering of the rows and columns, which is arbitrary. However, if one chooses the set of independent flux variables, i.e. columns to be skipped in the reduction process, the same reduced row echelon form of the matrix is found irregardless of the original ordering of the rows and columns. For this to be true, the columns corresponding to the chosen set must be linearly independent. When a viable set of independent flux variables is unknown or only partially known beforehand, the GJE routine implemented in SympyCore will choose the remaining independent flux variables to complete the matrix reduction process.

### Flux analysis in vivo

One of the most challenging tasks for the analysis of fluxes *in vivo *is intracellular compartmentation. There are several levels of compartmentation that ought to be taken into account in a large scale metabolic model. They range from the organ level to the sub-cellular level. The genome-scale metabolic models used in this text [10] are typical in that they are compartmentalized into standard intracellular compartments separated by membrane barriers, such as mitochondria. However, even smaller compartmental units exist such as submembrane space leading to the coupling between the K-ATP sensitive channel and creatine kinase [19], or intracellular diffusion barriers grouping ATPases and mitochondrial oxidative phosphorylation in cardiomyocytes [20-23], and the compartmentation of metabolites within enzyme systems [24]. These forms of compartmentation are often excluded from metabolic models. A genome-scale model that includes all such smaller compartmental units has yet to be formulated and will be larger. The symbolic GJE routine developed in this paper would be a suitable tool to analyze such large networks due to its efficiency.

Frequently, compartmentation can be analyzed by fully or partially decoupling the links between metabolites and reactions in the stoichiometric matrix. However, concentration gradients within the cell cannot be incorporated into a stoichiometric model. This form of compartmentation requires the use of reaction-diffusion models that take into account the three dimensional organization of the cell [25,26], and the development and application of specialized techniques such as the measurement of diffusion coefficient in the cell [27] and the use of kinetic measurements to estimate the diffusion restrictions partitioning the cell into compartments [22]. Thus the concentration gradients limit the application of stoichiometric modeling to the thermodynamic level.

Even without resorting to spatial modeling, the analysis of compartmentation remains challenging since more data is required to constrain the extra degrees of freedom introduced when splitting up metabolic pools. A recent organ level study of human brain [28] discusses the challenges of both composing an organ level compartmentalized model and obtaining the data required to constrain it. Our analysis of the example yeast network shows that each degree of freedom controls a local sub-networks of fluxes. By specifying intercompartmental fluxes to be part of the set of independent fluxes the influence of compartmentation may be characterized by a subset of variables making the analysis of compartmentation more straight forward.

Functional coupling within enzyme systems is often neglected in large scale metabolic models. When studying enzyme kinetics, it is often assumed that the distribution of the states of the enzyme remains stationary and is determined by the availability of metabolites. This assumption has been applied to study coupled enzyme systems [29] whose steady state is non-trivial since they may contain hundreds of transformations. When this assumption is made, individual mechanistic transformations can be treated in the same way as chemical reactions. The ability to choose some of these mechanistic transformations to be part of the set of independent fluxes would aid in the constraint process. It would also help one to incorporate enzyme mechanisms into larger stoichiometric models since the fluxes through the branches in the enzyme mechanism would be controlled by a subset of the independent variables and this subset would not influence remote regions of the metabolism.

Several approaches have been developed to study flux distributions *in vivo *without perturbing enzyme function. Notably, isotope labeling [30] and magnetization transfer [31]. The dynamic component of the labeling can be used to reveal compartmental effects such as the identification of barriers to metabolite transport. However this approach requires the use of optimization tools that must scan a high-dimensional space [30]. Recently, an improved optimization approach was developed that makes use of a flux coordinate system found using GJE [32]. Our GJE routine allows for the pre-selection of the independent variables, and it is anticipated that a well chosen flux coordinate system would further improve the application of this optimization procedure.

### Different representations of steady state solutions

The goals of constraint based flux analysis are currently pursued using an increasing number of complimentary approaches including extreme currents [33], extreme pathways [34], elementary modes [35,36], minimal generators [37], minimal metabolic behaviors [38], and other techniques [39]. In this paper we only applied symbolic GJE algorithm to carry out Metabolic Flux Analysis (MFA).

SympyCore can be extended by implementing the double description method [40] which is an integral part of Elementary Flux Mode Analysis (EFMA).

Although both MFA and EFMA provide solutions to the same steady state problem, comparing these solutions must take into account differences in the representations of the solutions and underlying assumptions in these methods. While MFA defines a subspace of steady state flux distributions then EFMA restricts this subspace by taking into account of irreversibility of certain reactions.

Within MFA, to represent a point in such a flux subspace, it is convenient to use a linear combination of the columns of the kernel of the stoichiometric matrix. Note that such a kernel is not unique: in the SVD approach the kernel depends on the ordering of reactions as they are used to compose the stoichiometric matrix; and in the symbolic GJE approach, the kernel depends on the initial choice of independent and dependent flux variables. Reaction irreversibilities convert to constraints on the coefficients of the linear combination. In the case of the SVD kernel, these constraints are difficult to interpret because of the convolved nature of the SVD coefficients: change of one coefficient will have effect to all fluxes. In the case of the GJE kernel, the coefficients are fluxes themselves (independent fluxes) and hence the constraints on the coefficients have a straightforward interpretation.

Within EFMA, it is mathematically more convenient to use convex polytope to represent the restricted part of the flux subspace because the conditions of reaction irreversibilities directly define the representation. This approach has given rise to the now widely used notation of elementary flux modes [41] and extreme pathways [34] that mathematically speaking are extreme rays of the convex polytope of thermodynamically feasible steady state flux distributions. It is interesting to note that in the case of pointed polytope the steady state flux distribution can be represented as a conical combination of elementary flux modes. While the elementary flux modes are uniquely determined then different combinations of elementary flux modes may define the same steady state solutions. This is orthogonal to kernel based representations: steady state solutions can be represented via different kernels but when fixing a kernel then the linear combination of its columns uniquely defines the flux distribution.

## Conclusions

A symbolic GJE routine was developed within SympyCore [11] to efficiently calculate the steady state flux distribution of genome-scale metabolic networks.

Constraints can be applied directly to each independent flux. The independent flux variables can be specified in the symbolic GJE routine to match the measured data available. In addition, it was demonstrated that knowledge regarding dependent flux variables can be used to find limits on the possible ranges of independent flux variables.

We found that independent fluxes influence only specific portions of the metabolism and sub-networks can be identified from the GJE kernel matrix. This property has potentially far reaching implications for the physical interpretation of steady metabolism in genome-scale metabolic networks.

Note that usage of the symbolic GJE routine does not introduce numerical errors while numerical SVD routines do. We estimated the relative flux error introduced by the numerical SVD routine and concluded that the numerical errors are insignificant for biological applications and confirm the numerical robustness of the SVD routine. Both numerical SVD and symbolic GJE routines are equivalent with respect to computation time, however, the memory consumed by numerical SVD routine increases two times faster than that of the symbolic GJE routine using sparse data structures.

The main arguments for using symbolic GJE routine for analyzing large metabolic networks are memory efficiency, numerical robustness, freedom of choosing different sets of independent fluxes, and the ability to define sub-networks.

Our results show that symbolic implementation of relevant algorithms are competitive with highly efficient numerical algorithms when taking into account the inherit sparsity of genome-scale metabolic networks.

## Methods

In this section we present two alternative procedures to obtain steady state solutions of possibly large under-determined metabolic networks. The first approach uses a symbolic GJE algorithm that guarantees exact solutions and the second approach uses SVD implemented in a numerical algorithm that ought to give better performance. In addition, we describe a method for applying constraints to the steady state solution.

### Statement of the steady state problem

Every chemical reaction and thus reaction system has the strict requirement of conservation of mass. A system of mass balances around each species has the form:(1)

where  is a length m vector of the time derivative for each mass density of metabolic species, **N **is the m × n stoichiometric matrix that links metabolites to their reactions via stoichiometry, and ***ν ***is a length n vector that describes the flux through each reaction. For a system at steady state with n reactions and m species, the system of chemical reactions becomes:(2)

The number of flux variables that need to be specified to calculate a viable steady state is f = n - r where r is the rank of **N**. Let us denote the vectors of dependent and independent flux variables as ***ν***_dep _and ***ν***_indep _of length r and f, respectively. Then with a n × n permutation matrix **P **that reorders the columns of **N **such that columns corresponding to dependent flux variables appear earliest, the steady state Equation (2) reads(3)

where  and **N **= [**N**_1 _**N**_2_] **P**^T^. Clearly, when the m × r matrix **N**_1 _is regular (m = r and det **N**_1 _≠ 0), the relation between ***ν***_dep _and ***ν***_indep _vectors can be computed directly:(4)

However, for many metabolic networks the stoichiometric matrix **N **may contain linearly dependent rows (r < m). In addition, ***ν***_indep _or **P **are not known in advance.

In the following we consider two methods based on GJE and SVD procedures that solve Equation (2) for the relation between dependent and independent flux variables: The general solution is written as:(5a)(5b)

We identify **R **as a kernel of the steady state solution where the columns are flux basis vectors and ***ν***_indep _are flux coordinates.

### Solving the steady state problem via GJE

Solving the steady state problem via GJE is based on transforming the stoichiometric matrix **N **to a row-echelon form **N**^GJE ^where all columns corresponding to dependent flux variables would have exactly one nonzero element and Equation (5) can be easily composed ( is identity matrix and hence ). The column permutation matrix **P **is constructed during the GJE process while applying the leading row and column selection rules (pivot element selection). One of the advantage of using GJE is that it allows one to influence the pivot element selection rules so that a preferred flux basis for the system will be obtained. If the selected flux variables cannot form a basis, the routine will move one or more of the preselected independent variables to become dependent.

Note that in numerical GJE algorithms the typical leading row and column selection rule consists of choosing a pivot element with largest absolute value for maximal numerical stability. Symbolic GJE algorithms that calculate in fractions avoid numerical rounding errors and can implement more optimal selection rules that take into account the sparsity of the system. In SympyCore [11] the leading row and column selection rule consists of choosing such a pivot element that minimizes the number of row operations for minimal computation time.

### Solving the steady state problem via SVD

Solving the steady state problem via SVD is based on decomposing the stoichiometric matrix **N **into a dot product of three matrices:(6)

where **u**, **v **= [**V**_im _**V**_ker_] are orthogonal matrices and ***σ ***is a diagonal matrix with nonzero values on the diagonal. The solution to the steady state Equation (2) is(7)

where ***α ***is a f vector of arbitrary parameters. Note that the SVD approach does not provide a numerically reliable and efficient way to determine the vectors of dependent and independent flux variables and in the following we use these in the form of the permutation matrix **P **found from the GJE approach:(8)

which gives Equation (5b):(9)

where **V**_indep _is a regular f × f matrix.

### Processing and analysis of metabolic networks

SBML models of metabolic networks were obtained from the BiGG database [42]. During the parsing all floating point numbers were converted to fractional numbers. All species that did not participate in any reactions were excluded. Species that are appear as both a reactant and product, i.e. in polymerization reactions, were removed from the list of reactants, and an additional reaction transporting this species across the system boundary was added.

Each metabolic network was transformed into open form using the following rule: if a species participated in exactly one reaction, a reaction transporting this species across the system boundary was added. As an alternative rule used in the example yeast network, if all transport reactions out of the system are known, then transformation to open form is accomplished by removing rows for the species that are external to the system.

Both of these approaches result in equivalent steady state solutions because adding extra reactions extends linear pathways that each contain a species that exits the system. Both approaches were applied to the example yeast network: external species were removed to calculate the flux distribution in Figure 3 and additional file 2: yeast_example.pdf while the algorithm to add extra transport reactions was used to calculate the values in Table 1.

### Composing the example yeast network

The example yeast network given in Figure 3 was manually composed for analyzing carbon isotope dynamics, and thus excludes metabolites that do not participate in carbon rearrangement, i.e. cofactors. To simplify the model, Carbon 3 of histidine (by InChI carbon number) was assumed to come from bicarbonate, and not Carbon 2 of ATP. Similarly, Carbon 1 of methionine was also assumed to come from bicarbonate, and not 5-Methyltetrahydropteroyltri-L-glutamate. In addition, the glyoxylate cycle and thus the third pathway for producing glycine was removed. All relevant details of the network including metabolite abbreviations, reaction definitions, the steady state solution, and substituted flux values used to constrain the system are given in additional file 2: yeast_example.pdf.

The example yeast network makes use of fictitious metabolites that link the stoichiometry of coupled reactions. The three pentose phosphate pathway reactions are broken into two parts each linked with a fictitious metabolite that represents the carbon skeleton that is broken off of one metabolite in the first step and transferred to the next. In the additional file 2: yeast_example.pdf fictitious metabolite names start with either a capital X, Y, or Z, followed by a lower case Greek number indicating the number of carbons they contain followed by a section indicating their use. This latter section is either the yeast enzyme they participate in, the code for the metabolite they are derived from, or GOG indicating the transfer of glutamate to 2-Oxoglutarate.

### Applying constraints to the steady state solution

The GJE routine provides a flux based coordinate system to describe the steady state flux space while SVD provides an orthogonal coordinate system. When specifying a flux value that is part of a flux coordinate system, one dimension from the steady state flux space is removed.

Many different sets of independent flux variables can form a coordinate system for the steady state flux space. The GJE routine allows the researcher to specify which flux variables forms a flux coordinate system and thus can match the choice of coordinate system with the experimental data available. The basis vectors formed from a flux coordinate system are often sparse and tend to span connected portions of the metabolism.

Let us assume a relation between dependent and independent flux variables as given in Equation (5). In addition to that, let us assume some constraining knowledge about the dependent variables, for example, the flux positivity for irreversible reactions:  for some *i *∈ [1; *r*]. The problem being solved is how the constraints on ***ν***_dep _constrain the independent flux variables ***ν ***_indep_. The goal is to determine how the steady state flux space is bounded. This is useful for many techniques used to analyze the properties of metabolic networks, for example in optimization procedures that must scan the steady state flux space while avoiding regions that are not feasible [32].

To find the constraints for ***ν ***_indep_, we set up the following system:(10a)(10b)(10c)

where g × f matrix **G **and g vector **b **define g measured data constraints for ***ν***_indep_; q × r matrix **Q **that defines q positivity constraints for ***ν***_dep_. The system in Equation (10) defines a convex polytope and due to the constraining parts it is redundant. The redundancy can be removed by using the following geometric computation algorithm: solve the vertex enumeration problem for the convex polytope defined by Equation (10) and then using the obtained vertexes and rays solve the facet enumeration problem. The solution to the facet enumeration problem is a set of inequalities that has no redundancies and defines the same convex polytope as Equation (10). Note that the intermediate result of the vertex enumeration problem (polytope vertexes and rays) provides convenient information to volume scanning applications.

### Computational software and error analysis

The GJE results of this paper are obtained using a Python package SympyCore [11] that implements both memory and processor efficient sparse matrix structures and manipulation algorithms. For solving the steady state problem we are using the symbolic matrix object method get_gauss_jordan_elimination_operations that allows one to specify the list of preferred leading columns (that is, the preferred list of dependent flux variables) for the GJE algorithm and after applying the GJE process the method returns a matrix object that is in row-echelon form. In addition to that, the method returns also a list of all applied row operations that can be later efficiently applied to other matrix objects. This feature is especially useful for adding extra columns to a stoichiometric matrix and then applying GJE process without the need to recompute the row-echelon form of the original matrix. One could use this to add transport reactions to a metabolic network during the constraint process.

The SVD results of this paper were obtained using a Python package NumPy [43] that provides a function numpy.linalg.svd for computing SVD of an array object. NumPy was built with LAPACK and ATLAS (version 3.8.3) libraries that provide a state-of-the-art routine (dgesdd) for computing SVD.

Since the results obtained with the symbolic GJE routine are correct and the results of the numerical SVD routine contain numerical rounding errors then in the error analysis we are using maximal relative flux error(11)

where  and  are matrix elements in Equation (5) obtained with GJE and SVD routines, respectively. Note that ε_SVD _characterizes relative errors in dependent flux variables introduced by the numerical SVD routine.

For solving vertex and facet enumeration problems we use a Python package pycddlib [44], a wrapper of the cddlib (version 094g) that implements the double description method [40].

The Python scripts used for computing the results are available in SympyCore [45]. The performance timings were obtained on a Ubuntu Linux dual-core (AMD Phenom(tm) II X2 550) computer with 4GB RAM.

## Authors' contributions

DS and PP developed the GJE technique with DS providing chemical insight and PP providing mathematical insight. DS composed the example yeast network and analyzed constraints. All authors contributed to the text and approved the content of the final manuscript.

## Supplementary Material

Additional file 1 **SBML model of the example yeast network**. This file is marked up in SBML and contains all of the reactions of the example yeast network. Click here for file

Additional file 2 **SBML model details**. This is a PDF file that summarizes the details of the model given in additional file 1: yeast_example.xml and presents all calculated results. Click here for file
