# Supplementary material for: Symbolic flux analysis for genome-scale metabolic networks
Source: BMC Syst Biol. 2011 May 23;5:81. doi: 10.1186/1752-0509-5-81 (PMC3130677; doi:10.1186/1752-0509-5-81)
Supplement: Additional file 2 — SBML model details. This is a PDF file that summarizes the details of the model given in additional file 1: yeast_example.xml and presents all calculated results. [file 1752-0509-5-81-S2.PDF]

Additional file 2 for the paper entitled:
Symbolic flux analysis for genome-scale metabolic networks

David W. Schryer[1], Marko Vendelin[1], Pearu Peterson*[1]

[1]Laboratory of Systems Biology, Institute of Cybernetics at Tallinn University of Technology, Akadeemia tee 21, 12618 Tallinn, Estonia

Email: David W. Schryer - david@sysbio.ioc.ee; Marko Vendelin - markov@sysbio.ioc.ee; Pearu Peterson*- pearu@sysbio.ioc.ee;

*Corresponding author

Contents

1 Introduction **1**

2 Contents of the SBML model of the example yeast network **2**
2.1 Definition of all species 2
2.2 Definition of all reactions 4

3 Symbolic solution of the steady state problem **9**
3.1 Flux relations 9
3.2 Constraints 13

4 Values of independent fluxes used to generate Figure 1. **15**

5 Symbolic solution of the steady state problem with measured values **16**
5.1 Flux relations 16
5.2 Constraints 19

1 Introduction

This document is a supplementary material to the above article and acts as a companion to the SBML file provided as additional file 1: yeast_example.xml. The content of this document is generated from the SBML file and acts as a convenient view of the model of yeast central metabolism. The composition of the model is discussed in the main text.

2 Contents of the SBML model of the example yeast network

2.1 Definition of all species

Legend: m - mitochondria, c - cytosol, x - external.

Symbol	Compartments	Description
AA	c, m	C00084 Acetaldehyde
AC	c, m	C00033 Acetate
AH	c	C01077 O-Acetyl-L-homoserine
ALA	c, m, x	C00041 L-Alanine
AN	c	C00108 Anthranilate
ARG	c, x	C00062 L-Arginine
AS	c	C03406 N-(L-Arginino)succinate
ASN	c, x	C00152 L-Asparagine
ASP	c, m, x	C00049 L-Aspartate
AcCoA	c, m, x	C00024 Acetyl-CoA
C	c	C00327 L-Citrulline
CH	c	C00251 Chorismate
CI	m	C00158 Citrate/Isocitrate
CO2	c, m, x	C00011 Carbon dioxide
CP	c	C00169 Carbamoyl phosphate
EivP	c	C00279 D-Erythrose 4-phosphate
FU	c, m	C00122 Fumarate
FviP	c, x	C05345 β-D-Fructose 6-phosphate
GL	c, x	C00031 D-Glucose
GLN	c, m, x	C00064 L-Glutamine
GLU	c, m, x	C00025 L-Glutamate
GLY	c, x	C00037 Glycine
GP	c	C00111 Glycerone phosphate
GiiiP	c, x	C00118 D-Glyceraldehyde 3-phosphate
GviP	c, x	C00668 α-D-Glucose 6-phosphate
HC	c	C00155 L-Homocysteine
HCO3-	c, m	C00288 Bicarbonate
HIS	c, x	C00135 L-Histidine
I	c	C00463 Indole
ILE	c, m, x	C00407 L-Isoleucine
IOS	c, m	C04236 (2S)-2-Isopropyl-3-oxosuccinate
IP	c	C03506 Indoleglycerol phosphate
LEU	c, m, x	C00123 L-Leucine
LYS	c, x	C00047 L-Lysine
MA	m	C00149 Malate
MET	c, x	C00073 L-Methionine
MO	c, m	C00141 3-Methyl-2-oxobutanoic acid

table continues ...

Table 1: (continued)

Symbol	Compartments	Description
OA	c, m, x	C00036 Oxaloacetate
OAG	c	C05533 Oxaloglutarate
OG	c, m	C00026 2-Oxoglutarate
OR	c, m	C00077 L-Ornithine
PEP	c	C00074 Phosphoenolpyruvate
PHE	c, x	C00079 L-Phenylalanine
PRO	c, x	C00148 L-Proline
PY	c, m	C00022 Pyruvate
PvRA	c	C04302 N-(5-Phospho-β-D-ribosyl)anthranilate
RLvP	c	C00199 D-Ribulose 5-phosphate
RvP	c, x	C00117 D-Ribose 5-phosphate
SER	c, x	C00065 L-Serine
SU	m	C00042 Succinate
SUCoA	m	C00091 Succinyl-CoA
SiiiP	c	C03175 Shikimate 3-phosphate
SviiP	c	C05382 D-Sedoheptulose 7-phosphate
THR	c, m, x	C00188 L-Threonine
TRP	c, x	C00078 L-Tryptophan
TYR	c, x	C00082 L-Tyrosine
VAL	c, m, x	C00183 L-Valine
XvP	c	C00231 D-Xylulose 5-phosphate
XviCPA	c	Six carbon analogue used in the CPA reaction.
YiGOG	c, m	One carbon analogue used in GLU-OG stoichiometry.
YiiXvP	c	Intermediate product of reaction R01830 (TKL1 or TKL2)
ZiiPY	m	Part of PY used in ILE, VAL, and LEU biosynthesis.
ZiiXvP	c	Intermediate product of reaction R01641 (TKL1 or TKL2)
ZiiiFviP	c	Intermediate product of reaction R01827 (TAL1)
ZivALT	c, m	Used to link the stoichiometry of the ALT reaction.
ZvAAT	c, m	Used to link the stoichiometry of the AAT1 and AAT2 reactions.
ZviGOG	c, m	Six carbon analogue used in GLU-OG stoichiometry.

2.2 Definition of all reactions

Gene		Reaction/flux	
	**Cytosolic reactions:**		
	AAo	$\xrightleftharpoons{\nu_{\text{AAo\_ACo}}}$	ACo
	ACo	$\xrightleftharpoons{\nu_{\text{ACo\_AcCoAo}}}$	AcCoAo
	BCo + GLNo	$\xrightleftharpoons{\nu_{\text{BCo\_CPo\_01}}}$	XviCPAo
	XviCPAo	$\xrightleftharpoons{\nu_{\text{BCo\_CPo\_02}}}$	CPo + GLUo
	AHo	$\xrightleftharpoons{\nu_{\text{B\_AC\_01}}}$	ACo + HCo
	ASPo	$\xrightleftharpoons{\nu_{\text{B\_ASP\_ASN}}}$	ASNo
	ASPo	$\xrightleftharpoons{\nu_{\text{B\_ASP\_THR}}}$	THRo
	ASPo + AcCoAo	$\xrightleftharpoons{\nu_{\text{B\_AcCoA\_01}}}$	AHo
	GLUo	$\xrightleftharpoons{\nu_{\text{B\_GLU\_GLN}}}$	GLNo
	GLUo	$\xrightleftharpoons{\nu_{\text{B\_GLU\_PRO}}}$	PROo
	SERo	$\xrightarrow{\nu_{\text{B\_GiiiP\_GLY}}}$	CO_2 + GLYo
	GiiiPo	$\xrightleftharpoons{\nu_{\text{B\_GiiiP\_SER}}}$	SERo
	AcCoAo + OGo	$\xrightleftharpoons{\nu_{\text{B\_LYS\_C1}}}$	OAGo
	OAGo	$\xrightarrow{\nu_{\text{B\_LYS\_C2}}}$	CO_2 + LYSo
	BCo + HCo	$\xrightleftharpoons{\nu_{\text{B\_OA\_AcCoA\_MET}}}$	METo
	BCo + OAo	$\xrightleftharpoons{\nu_{\text{B\_OAo\_ASP\_01}}}$	ZvAATo
	ZvAATo	$\xrightleftharpoons{\nu_{\text{B\_OAo\_ASP\_02}}}$	ASPo + YiGOGo
	OGo	$\xrightleftharpoons{\nu_{\text{B\_OG\_GLU\_C}}}$	GLUo
	PEPo + SiiiPo	$\xrightleftharpoons{\nu_{\text{B\_PEP\_01}}}$	CHo
	EivPo + PEPo	$\xrightleftharpoons{\nu_{\text{B\_PEP\_EivP\_01}}}$	SiiiPo
	CHo	$\xrightarrow{\nu_{\text{B\_PEP\_EivP\_PHE}}}$	CO_2 + PHEo
	CHo	$\xrightleftharpoons{\nu_{\text{B\_PEP\_EivP\_TRP\_01}}}$	ANo + PYo
	ANo + RvPo	$\xrightleftharpoons{\nu_{\text{B\_PEP\_EivP\_TRP\_02}}}$	PvRAo
	PvRAo	$\xrightarrow{\nu_{\text{B\_PEP\_EivP\_TRP\_03}}}$	CO_2 + IPo
	IPo	$\xrightleftharpoons{\nu_{\text{B\_PEP\_EivP\_TRP\_04}}}$	GiiiPo + Io
	Io + SERo	$\xrightleftharpoons{\nu_{\text{B\_PEP\_EivP\_TRP\_05}}}$	TRPo
	CHo	$\xrightarrow{\nu_{\text{B\_PEP\_EivP\_TYR}}}$	CO_2 + TYRo
	BCo + PYo	$\xrightleftharpoons{\nu_{\text{B\_PY\_ALA\_C1}}}$	ZivALTo
	ZivALTo	$\xrightleftharpoons{\nu_{\text{B\_PY\_ALA\_C2}}}$	ALAo + YiGOGo
	AcCoAo + MOo	$\xrightleftharpoons{\nu_{\text{B\_PY\_AcCoA\_C}}}$	IOSo
	IOSo	$\xrightarrow{\nu_{\text{B\_PY\_AcCoA\_LEU\_C}}}$	CO_2 + LEUo
	MOo	$\xrightleftharpoons{\nu_{\text{B\_PY\_VAL\_C}}}$	VALo
	BCo + RvPo	$\xrightleftharpoons{\nu_{\text{B\_RvP\_HIS}}}$	HISo

table continues ...

Table 2: (continued)

Gene	Reaction/flux		
	THRo	$\xrightleftharpoons{\nu_{\text{B\_THR\_GLY}}}$	ACo + GLYo
	CO_2	$\xrightleftharpoons{\nu_{\text{CD\_BC}}}$	BCo
	FviPo	$\xrightleftharpoons{\nu_{\text{FviP\_GP\_GiiiP}}}$	GPo + GiiiPo
	GLUo + YiGOGo	$\xrightleftharpoons{\nu_{\text{GLUo\_OGo\_01}}}$	ZviGOGo
	ZviGOGo	$\xrightleftharpoons{\nu_{\text{GLUo\_OGo\_02}}}$	BCo + OGo
	GLo	$\xrightleftharpoons{\nu_{\text{GL\_GviP}}}$	GviPo
	GPo	$\xrightleftharpoons{\nu_{\text{GP\_GiiiP}}}$	GiiiPo
	GiiiPo	$\xrightleftharpoons{\nu_{\text{GiiiP\_PEP}}}$	PEPo
	GviPo	$\xrightleftharpoons{\nu_{\text{GviP\_FviP}}}$	FviPo
	GviPo	$\xrightarrow{\nu_{\text{GviP\_RLvP}}}$	CO_2 + RLvPo
	OAo	$\xrightarrow{\nu_{\text{OA\_PEP}}}$	CO_2 + PEPo
	PEPo	$\xrightleftharpoons{\nu_{\text{PEP\_PY}}}$	PYo
	GiiiPo + ZiiiFviPo	$\xrightleftharpoons{\nu_{\text{PPP\_FviP\_EivP\_SviiP\_GiiiP\_01}}}$	FviPo
	SviiPo	$\xrightleftharpoons{\nu_{\text{PPP\_FviP\_EivP\_SviiP\_GiiiP\_02}}}$	EivPo + ZiiiFviPo
	FviPo	$\xrightleftharpoons{\nu_{\text{PPP\_FviP\_GiiiP\_XvP\_EivP\_01}}}$	EivPo + YiiXvPo
	GiiiPo + YiiXvPo	$\xrightleftharpoons{\nu_{\text{PPP\_FviP\_GiiiP\_XvP\_EivP\_02}}}$	XvPo
	RvPo + ZiiXvPo	$\xrightleftharpoons{\nu_{\text{PPP\_SviiP\_GiiiP\_XvP\_RvP\_01}}}$	SviiPo
	XvPo	$\xrightleftharpoons{\nu_{\text{PPP\_SviiP\_GiiiP\_XvP\_RvP\_02}}}$	GiiiPo + ZiiXvPo
	PYo	$\xrightarrow{\nu_{\text{PYo\_AAo}}}$	AAo + CO_2
	BCo + PYo	$\xrightleftharpoons{\nu_{\text{PYo\_OAo}}}$	OAo
	RLvPo	$\xrightleftharpoons{\nu_{\text{RLvP\_RvP}}}$	RvPo
	ARGo	$\xrightarrow{\nu_{\text{US\_ARG\_OR}}}$	CO_2 + ORo
	ASo	$\xrightleftharpoons{\nu_{\text{US\_AS\_FU\_ARG}}}$	ARGo + FUo
	ASPo + Co	$\xrightleftharpoons{\nu_{\text{US\_C\_ASP\_AS}}}$	ASo
	CPo + ORo	$\xrightleftharpoons{\nu_{\text{US\_OR\_C}}}$	Co
	XvPo	$\xrightleftharpoons{\nu_{\text{XvP\_RLvP}}}$	RLvPo
	**Transport reactions:**		
	ALAo	$\xrightarrow{\nu_{\text{ALA}_{\text{out}}}}$	ALAx
	ARGo	$\xrightarrow{\nu_{\text{ARG}_{\text{out}}}}$	ARGx
	ASNo	$\xrightarrow{\nu_{\text{ASN}_{\text{out}}}}$	ASNx
	ASPo	$\xrightarrow{\nu_{\text{ASP}_{\text{out}}}}$	ASPx
	AcCoAo	$\xrightarrow{\nu_{\text{AcCoA}_{\text{out}}}}$	AcCoAx
	CO_2	$\xrightarrow{\nu_{CO_2,\text{out}}}$	CO_2

table continues ...

Table 2: (continued)

Gene	Reaction/flux		
	FviPo	$\xrightarrow{\nu_{\text{FviP}_{\text{out}}}}$	FviPx
	GLNo	$\xrightarrow{\nu_{\text{GLN}_{\text{out}}}}$	GLNx
	GLUo	$\xrightarrow{\nu_{\text{GLU}_{\text{out}}}}$	GLUx
	GLYo	$\xrightarrow{\nu_{\text{GLY}_{\text{out}}}}$	GLYx
	GLx	$\xrightarrow{\nu_{\text{GL}_{\text{in}}}}$	GLo
	GiiiPo	$\xrightarrow{\nu_{\text{GiiiP}_{\text{out}}}}$	GiiiPx
	GviPo	$\xrightarrow{\nu_{\text{GviP}_{\text{out}}}}$	GviPx
	HISo	$\xrightarrow{\nu_{\text{HIS}_{\text{out}}}}$	HISx
	ILEo	$\xrightarrow{\nu_{\text{ILE}_{\text{out}}}}$	ILEx
	LEUo	$\xrightarrow{\nu_{\text{LEU}_{\text{out}}}}$	LEUx
	LYSo	$\xrightarrow{\nu_{\text{LYS}_{\text{out}}}}$	LYSx
	METo	$\xrightarrow{\nu_{\text{MET}_{\text{out}}}}$	METx
	OAo	$\xrightarrow{\nu_{\text{OA}_{\text{out}}}}$	OAx
	PHEo	$\xrightarrow{\nu_{\text{PHE}_{\text{out}}}}$	PHEx
	PROo	$\xrightarrow{\nu_{\text{PRO}_{\text{out}}}}$	PROx
	RvPo	$\xrightarrow{\nu_{\text{RvP}_{\text{out}}}}$	RvPx
	SERo	$\xrightarrow{\nu_{\text{SER}_{\text{out}}}}$	SERx
	THRo	$\xrightarrow{\nu_{\text{THR}_{\text{out}}}}$	THRx
	TRPo	$\xrightarrow{\nu_{\text{TRP}_{\text{out}}}}$	TRPx
	TYRo	$\xrightarrow{\nu_{\text{TYR}_{\text{out}}}}$	TYRx
	VALo	$\xrightarrow{\nu_{\text{VAL}_{\text{out}}}}$	VALx
	**Cytosolic-mitochondrial transport reactions:**		
	AAo	$\xrightleftharpoons{\nu_{\text{AAo\_AAm}}}$	AAm
	ALAm	$\xrightleftharpoons{\nu_{\text{ALAm\_ALAo}}}$	ALAo
	ASPm	$\xrightleftharpoons{\nu_{\text{ASPm\_ASPo}}}$	ASPo
	CO_2	$\xrightleftharpoons{\nu_{\text{CDm\_CDo}}}$	CO_2
	FUo	$\xrightleftharpoons{\nu_{\text{FUo\_FUm}}}$	FUm
	GLNo	$\xrightleftharpoons{\nu_{\text{GLNo\_GLNm}}}$	GLNm
	GLUm	$\xrightleftharpoons{\nu_{\text{GLUm\_GLUo}}}$	GLUo
	ILEm	$\xrightleftharpoons{\nu_{\text{ILEm\_ILEo}}}$	ILEo
	IOSm	$\xrightleftharpoons{\nu_{\text{IOSm\_IOSo}}}$	IOSo
	LEUm	$\xrightleftharpoons{\nu_{\text{LEUm\_LEUo}}}$	LEUo

table continues ...

Table 2: (continued)

Gene	Reaction/flux		
	MOm	$\xrightleftharpoons{\nu_{\text{MOm\_MOo}}}$	MOo
	OAo	$\xrightleftharpoons{\nu_{\text{OAo\_OAm}}}$	OAm
	OGo	$\xrightleftharpoons{\nu_{\text{OGo\_OGm}}}$	OGm
	ORm	$\xrightleftharpoons{\nu_{\text{ORm\_ORo}}}$	ORo
	PYo	$\xrightleftharpoons{\nu_{\text{PYo\_PYm}}}$	PYm
	THRo	$\xrightleftharpoons{\nu_{\text{THRo\_THRm}}}$	THRm
	VALm	$\xrightleftharpoons{\nu_{\text{VALm\_VALo}}}$	VALo
	**Mitochondrial reactions:**		
	AAm	$\xrightleftharpoons{\nu_{\text{AAm\_ACm}}}$	ACm
	ACm	$\xrightleftharpoons{\nu_{\text{ACm\_AcCoAm}}}$	AcCoAm
	GLNm + OGm	$\xrightleftharpoons{\nu_{\text{B\_GLT}}}$	2 GLUm
	GLUm	$\xrightleftharpoons{\nu_{\text{B\_GLU\_OR}}}$	ORm
	HCO_3^- + OAm	$\xrightleftharpoons{\nu_{\text{B\_OAm\_ASP\_01}}}$	ZvAATm
	ZvAATm	$\xrightleftharpoons{\nu_{\text{B\_OAm\_ASP\_02}}}$	ASPm + YiGOGm
	OGm	$\xrightleftharpoons{\nu_{\text{B\_OG\_GLU\_M}}}$	GLUm
	HCO_3^- + PYm	$\xrightleftharpoons{\nu_{\text{B\_PY\_ALA\_M1}}}$	ZivALTm
	ZivALTm	$\xrightleftharpoons{\nu_{\text{B\_PY\_ALA\_M2}}}$	ALAm + YiGOGm
	IOSm	$\xrightarrow{\nu_{\text{B\_PY\_AcCoA\_LEU\_M}}}$	CO_2 + LEUm
	AcCoAm + MOm	$\xrightleftharpoons{\nu_{\text{B\_PY\_AcCoA\_M}}}$	IOSm
	PYm	$\xrightarrow{\nu_{\text{B\_PY\_M1}}}$	CO_2 + ZiiPYm
	PYm + ZiiPYm	$\xrightleftharpoons{\nu_{\text{B\_PY\_M2}}}$	MOm
	THRm + ZiiPYm	$\xrightleftharpoons{\nu_{\text{B\_PY\_THR\_ILE}}}$	ILEm
	MOm	$\xrightleftharpoons{\nu_{\text{B\_PY\_VAL\_M}}}$	VALm
	GLUm + YiGOGm	$\xrightleftharpoons{\nu_{\text{GLUm\_OGm\_01}}}$	ZviGOGm
	ZviGOGm	$\xrightleftharpoons{\nu_{\text{GLUm\_OGm\_02}}}$	HCO_3^- + OGm
	CIm	$\xrightarrow{\nu_{\text{TCA\_CIm\_OGm}}}$	CO_2 + OGm
	FUm	$\xrightleftharpoons{\nu_{\text{TCA\_FUm\_MAm}}}$	MAm
	MAm	$\xrightleftharpoons{\nu_{\text{TCA\_MAm\_OAm}}}$	OAm
	MAm	$\xrightarrow{\nu_{\text{TCA\_MAm\_PYm}}}$	CO_2 + PYm
	AcCoAm + OAm	$\xrightleftharpoons{\nu_{\text{TCA\_OAm\_AcCoAm\_CIm}}}$	CIm
	OGm	$\xrightarrow{\nu_{\text{TCA\_OGm\_SUCoAm}}}$	CO_2 + SUCoAm
	PYm	$\xrightarrow{\nu_{\text{TCA\_PYm\_AcCoAm}}}$	AcCoAm + CO_2
	SUCoAm	$\xrightleftharpoons{\nu_{\text{TCA\_SUCoAm\_SUm}}}$	SUm

table continues ...

Table 2: (continued)

Gene	Reaction/flux
	SUm $\underset{}{\overset{\nu_{\mathrm{TCA\_SUm\_FUm}}}{\rightleftharpoons}}$ FUm

3 Symbolic solution of the steady state problem

3.1 Flux relations

Cytosolic flux relations:

$\nu_{\text{AAo\_ACo}} = \nu_{\text{AcCoA}_{\text{out}}} - \nu_{\text{B\_PY\_AcCoA\_LEU\_M}} - \nu_{\text{B\_THR\_GLY}} - \nu_{\text{IOSm\_IOSo}} + \nu_{\text{LEU}_{\text{out}}} + \nu_{\text{LYS}_{\text{out}}}$

$\nu_{\text{ACo\_AcCoAo}} = \nu_{\text{AcCoA}_{\text{out}}} - \nu_{\text{B\_PY\_AcCoA\_LEU\_M}} - \nu_{\text{IOSm\_IOSo}} + \nu_{\text{LEU}_{\text{out}}} + \nu_{\text{LYS}_{\text{out}}} + \nu_{\text{MET}_{\text{out}}}$

$\nu_{\text{BCo\_CPo\_01}} = \nu_{\text{US\_C\_ASP\_AS}}$

$\nu_{\text{BCo\_CPo\_02}} = \nu_{\text{US\_C\_ASP\_AS}}$

$\nu_{\text{B\_AC\_01}} = \nu_{\text{MET}_{\text{out}}}$

$\nu_{\text{B\_ASP\_ASN}} = \nu_{\text{ASN}_{\text{out}}}$

$\nu_{\text{B\_ASP\_THR}} = \nu_{\text{B\_THR\_GLY}} + \nu_{\text{ILE}_{\text{out}}} + \nu_{\text{THR}_{\text{out}}}$

$\nu_{\text{B\_AcCoA\_01}} = \nu_{\text{MET}_{\text{out}}}$

$\nu_{\text{B\_GLU\_GLN}} = \nu_{\text{B\_GLT}} + \nu_{\text{GLN}_{\text{out}}} + \nu_{\text{US\_C\_ASP\_AS}}$

$\nu_{\text{B\_GLU\_PRO}} = \nu_{\text{PRO}_{\text{out}}}$

$\nu_{\text{B\_GiiiP\_GLY}} = -\nu_{\text{B\_THR\_GLY}} + \nu_{\text{GLY}_{\text{out}}}$

$\nu_{\text{B\_GiiiP\_SER}} = -\nu_{\text{B\_THR\_GLY}} + \nu_{\text{GLY}_{\text{out}}} + \nu_{\text{SER}_{\text{out}}} + \nu_{\text{TRP}_{\text{out}}}$

$\nu_{\text{B\_LYS\_C1}} = \nu_{\text{LYS}_{\text{out}}}$

$\nu_{\text{B\_LYS\_C2}} = \nu_{\text{LYS}_{\text{out}}}$

$\nu_{\text{B\_OA\_AcCoA\_MET}} = \nu_{\text{MET}_{\text{out}}}$

$\nu_{\text{B\_OAo\_ASP\_01}} = \nu_{\text{ASN}_{\text{out}}} + \nu_{\text{ASP}_{\text{out}}} - \nu_{\text{ASPm\_ASPo}} + \nu_{\text{B\_THR\_GLY}} + \nu_{\text{ILE}_{\text{out}}} + \nu_{\text{MET}_{\text{out}}} + \nu_{\text{THR}_{\text{out}}} + \nu_{\text{US\_C\_ASP\_AS}}$

$\nu_{\text{B\_OAo\_ASP\_02}} = \nu_{\text{ASN}_{\text{out}}} + \nu_{\text{ASP}_{\text{out}}} - \nu_{\text{ASPm\_ASPo}} + \nu_{\text{B\_THR\_GLY}} + \nu_{\text{ILE}_{\text{out}}} + \nu_{\text{MET}_{\text{out}}} + \nu_{\text{THR}_{\text{out}}} + \nu_{\text{US\_C\_ASP\_AS}}$

$\nu_{\text{B\_OG\_GLU\_C}} = \nu_{\text{ALA}_{\text{out}}} + \nu_{\text{ASN}_{\text{out}}} + \nu_{\text{ASP}_{\text{out}}} - \nu_{\text{ASPm\_ASPo}} - \nu_{\text{B\_PY\_ALA\_M1}} + \nu_{\text{B\_THR\_GLY}} + \nu_{\text{ILE}_{\text{out}}} - \nu_{\text{LYS}_{\text{out}}} + \nu_{\text{MET}_{\text{out}}} - \nu_{\text{OGo\_OGm}} + \nu_{\text{THR}_{\text{out}}} + \nu_{\text{US\_C\_ASP\_AS}}$

$\nu_{\text{B\_PEP\_01}} = \nu_{\text{PHE}_{\text{out}}} + \nu_{\text{TRP}_{\text{out}}} + \nu_{\text{TYR}_{\text{out}}}$

$\nu_{\text{B\_PEP\_EivP\_01}} = \nu_{\text{PHE}_{\text{out}}} + \nu_{\text{TRP}_{\text{out}}} + \nu_{\text{TYR}_{\text{out}}}$

$\nu_{\text{B\_PEP\_EivP\_PHE}} = \nu_{\text{PHE}_{\text{out}}}$

$\nu_{\text{B\_PEP\_EivP\_TRP\_01}} = \nu_{\text{TRP}_{\text{out}}}$

$\nu_{\text{B\_PEP\_EivP\_TRP\_02}} = \nu_{\text{TRP}_{\text{out}}}$

$\nu_{\text{B\_PEP\_EivP\_TRP\_03}} = \nu_{\text{TRP}_{\text{out}}}$

$\nu_{\text{B\_PEP\_EivP\_TRP\_04}} = \nu_{\text{TRP}_{\text{out}}}$

$\nu_{\text{B\_PEP\_EivP\_TRP\_05}} = \nu_{\text{TRP}_{\text{out}}}$

$\nu_{\text{B\_PEP\_EivP\_TYR}} = \nu_{\text{TYR}_{\text{out}}}$

$\nu_{\text{B\_PY\_ALA\_C1}} = \nu_{\text{ALA}_{\text{out}}} - \nu_{\text{B\_PY\_ALA\_M1}}$

$\nu_{\text{B\_PY\_ALA\_C2}} = \nu_{\text{ALA}_{\text{out}}} - \nu_{\text{B\_PY\_ALA\_M1}}$

$\nu_{\text{B\_PY\_AcCoA\_C}} = -\nu_{\text{B\_PY\_AcCoA\_LEU\_M}} - \nu_{\text{IOSm\_IOSo}} + \nu_{\text{LEU}_{\text{out}}}$

$\nu_{\text{B\_PY\_AcCoA\_LEU\_C}} = -\nu_{\text{B\_PY\_AcCoA\_LEU\_M}} + \nu_{\text{LEU}_{\text{out}}}$

$\nu_{\text{B\_PY\_VAL\_C}} = -\nu_{\text{B\_PY\_VAL\_M}} + \nu_{\text{VAL}_{\text{out}}}$

$\nu_{\text{B\_RvP\_HIS}} = \nu_{\text{HIS}_{\text{out}}}$

$\nu_{\text{CD\_BC}} = \nu_{\text{ARG}_{\text{out}}} + \nu_{\text{ASN}_{\text{out}}} + \nu_{\text{ASP}_{\text{out}}} + \nu_{\text{B\_THR\_GLY}} + \nu_{\text{GLN}_{\text{out}}} + \nu_{\text{GLU}_{\text{out}}} + \nu_{\text{HIS}_{\text{out}}} + \nu_{\text{ILE}_{\text{out}}} + \nu_{\text{LYS}_{\text{out}}} + 2\nu_{\text{MET}_{\text{out}}} + \nu_{\text{OA\_PEP}} + \nu_{\text{OA}_{\text{out}}} + \nu_{\text{PRO}_{\text{out}}} + \nu_{\text{TCA\_MAm\_PYm}} + \nu_{\text{THR}_{\text{out}}} + \nu_{\text{US\_C\_ASP\_AS}}$

$\nu_{\text{FviP\_GP\_GiiiP}} = -\nu_{\text{FviP}_{\text{out}}} + \nu_{\text{GL}_{\text{in}}} - 1/3\nu_{\text{GviP\_RLvP}} - \nu_{\text{GviP}_{\text{out}}} - 2/3\nu_{\text{HIS}_{\text{out}}} - 1/3\nu_{\text{PHE}_{\text{out}}} - 2/3\nu_{\text{RvP}_{\text{out}}} - \nu_{\text{TRP}_{\text{out}}} - 1/3\nu_{\text{TYR}_{\text{out}}}$

$\nu_{\text{GLUo\_OGo\_01}} = \nu_{\text{ALA}_{\text{out}}} + \nu_{\text{ASN}_{\text{out}}} + \nu_{\text{ASP}_{\text{out}}} - \nu_{\text{ASPm\_ASPo}} - \nu_{\text{B\_PY\_ALA\_M1}} + \nu_{\text{B\_THR\_GLY}} + \nu_{\text{ILE}_{\text{out}}} + \nu_{\text{MET}_{\text{out}}} + \nu_{\text{THR}_{\text{out}}} + \nu_{\text{US\_C\_ASP\_AS}}$

$\nu_{\text{GLUo\_OGo\_02}} = \nu_{\text{ALA}_{\text{out}}} + \nu_{\text{ASN}_{\text{out}}} + \nu_{\text{ASP}_{\text{out}}} - \nu_{\text{ASPm\_ASPo}} - \nu_{\text{B\_PY\_ALA\_M1}} + \nu_{\text{B\_THR\_GLY}} + \nu_{\text{ILE}_{\text{out}}} + \nu_{\text{MET}_{\text{out}}} + \nu_{\text{THR}_{\text{out}}} + \nu_{\text{US\_C\_ASP\_AS}}$

$\nu_{\text{GL\_GviP}} = \nu_{\text{GL}_{\text{in}}}$

$\nu_{\text{GP\_GiiiP}} = -\nu_{\text{FviP}_{\text{out}}} + \nu_{\text{GL}_{\text{in}}} - 1/3\nu_{\text{GviP\_RLvP}} - \nu_{\text{GviP}_{\text{out}}} - 2/3\nu_{\text{HIS}_{\text{out}}} - 1/3\nu_{\text{PHE}_{\text{out}}} - 2/3\nu_{\text{RvP}_{\text{out}}} - \nu_{\text{TRP}_{\text{out}}} - 1/3\nu_{\text{TYR}_{\text{out}}}$

$\nu_{\text{GiiiP\_PEP}} = \nu_{\text{B\_THR\_GLY}} - 2\nu_{\text{FviP}_{\text{out}}} - \nu_{\text{GLY}_{\text{out}}} + 2\nu_{\text{GL}_{\text{in}}} - \nu_{\text{GiiiP}_{\text{out}}} - 1/3\nu_{\text{GviP\_RLvP}} - 2\nu_{\text{GviP}_{\text{out}}} - 5/3\nu_{\text{HIS}_{\text{out}}} - 4/3\nu_{\text{PHE}_{\text{out}}} - 5/3\nu_{\text{RvP}_{\text{out}}} - \nu_{\text{SER}_{\text{out}}} - 3\nu_{\text{TRP}_{\text{out}}} - 4/3\nu_{\text{TYR}_{\text{out}}}$

$\nu_{\text{GviP\_FviP}} = \nu_{\text{GL}_{\text{in}}} - \nu_{\text{GviP\_RLvP}} - \nu_{\text{GviP}_{\text{out}}}$

$\nu_{\text{PEP\_PY}} = \nu_{\text{B\_THR\_GLY}} - 2\nu_{\text{FviP}_{\text{out}}} - \nu_{\text{GLY}_{\text{out}}} + 2\nu_{\text{GL}_{\text{in}}} - \nu_{\text{GiiiP}_{\text{out}}} - 1/3\nu_{\text{GviP\_RLvP}} - 2\nu_{\text{GviP}_{\text{out}}} - 5/3\nu_{\text{HIS}_{\text{out}}} + \nu_{\text{OA\_PEP}} - 10/3\nu_{\text{PHE}_{\text{out}}} - 5/3\nu_{\text{RvP}_{\text{out}}} - \nu_{\text{SER}_{\text{out}}} - 5\nu_{\text{TRP}_{\text{out}}} - 10/3\nu_{\text{TYR}_{\text{out}}}$

$\nu_{\text{PPP\_FviP\_EivP\_SviiP\_GiiiP\_01}} = 1/3\nu_{\text{GviP\_RLvP}} - 1/3\nu_{\text{HIS}_{\text{out}}} + 1/3\nu_{\text{PHE}_{\text{out}}} - 1/3\nu_{\text{RvP}_{\text{out}}} + 1/3\nu_{\text{TYR}_{\text{out}}}$

$\nu_{\text{PPP\_FviP\_EivP\_SviiP\_GiiiP\_02}} = 1/3\nu_{\text{GviP\_RLvP}} - 1/3\nu_{\text{HIS}_{\text{out}}} + 1/3\nu_{\text{PHE}_{\text{out}}} - 1/3\nu_{\text{RvP}_{\text{out}}} + 1/3\nu_{\text{TYR}_{\text{out}}}$

$\nu_{\text{PPP\_FviP\_GiiiP\_XvP\_EivP\_01}} = -1/3\nu_{\text{GviP\_RLvP}} + 1/3\nu_{\text{HIS}_{\text{out}}} + 2/3\nu_{\text{PHE}_{\text{out}}} + 1/3\nu_{\text{RvP}_{\text{out}}} + \nu_{\text{TRP}_{\text{out}}} + 2/3\nu_{\text{TYR}_{\text{out}}}$

$\nu_{\text{PPP\_FviP\_GiiiP\_XvP\_EivP\_02}} = -1/3\nu_{\text{GviP\_RLvP}} + 1/3\nu_{\text{HIS}_{\text{out}}} + 2/3\nu_{\text{PHE}_{\text{out}}} + 1/3\nu_{\text{RvP}_{\text{out}}} + \nu_{\text{TRP}_{\text{out}}} + 2/3\nu_{\text{TYR}_{\text{out}}}$

$\nu_{\text{PPP\_SviiP\_GiiiP\_XvP\_RvP\_01}} = 1/3\nu_{\text{GviP\_RLvP}} - 1/3\nu_{\text{HIS}_{\text{out}}} + 1/3\nu_{\text{PHE}_{\text{out}}} - 1/3\nu_{\text{RvP}_{\text{out}}} + 1/3\nu_{\text{TYR}_{\text{out}}}$

$\nu_{\text{PPP\_SviiP\_GiiiP\_XvP\_RvP\_02}} = 1/3\nu_{\text{GviP\_RLvP}} - 1/3\nu_{\text{HIS}_{\text{out}}} + 1/3\nu_{\text{PHE}_{\text{out}}} - 1/3\nu_{\text{RvP}_{\text{out}}} + 1/3\nu_{\text{TYR}_{\text{out}}}$

$\nu_{\text{PYo\_OAo}} = \nu_{\text{ARG}_{\text{out}}} + \nu_{\text{ASN}_{\text{out}}} + \nu_{\text{ASP}_{\text{out}}} + \nu_{\text{B\_THR\_GLY}} + \nu_{\text{GLN}_{\text{out}}} + \nu_{\text{GLU}_{\text{out}}} + \nu_{\text{ILE}_{\text{out}}} + \nu_{\text{LYS}_{\text{out}}} + \nu_{\text{MET}_{\text{out}}} + \nu_{\text{OA\_PEP}} + \nu_{\text{OA}_{\text{out}}} + \nu_{\text{PRO}_{\text{out}}} + \nu_{\text{TCA\_MAm\_PYm}} + \nu_{\text{THR}_{\text{out}}}$

$\nu_{\text{RLvP\_RvP}} = 1/3\nu_{\text{GviP\_RLvP}} + 2/3\nu_{\text{HIS}_{\text{out}}} + 1/3\nu_{\text{PHE}_{\text{out}}} + 2/3\nu_{\text{RvP}_{\text{out}}} + \nu_{\text{TRP}_{\text{out}}} + 1/3\nu_{\text{TYR}_{\text{out}}}$

$\nu_{\text{US\_ARG\_OR}} = -\nu_{\text{ARG}_{\text{out}}} + \nu_{\text{US\_C\_ASP\_AS}}$

$\nu_{\text{US\_AS\_FU\_ARG}} = \nu_{\text{US\_C\_ASP\_AS}}$

$\nu_{\text{US\_OR\_C}} = \nu_{\text{US\_C\_ASP\_AS}}$

$\nu_{\text{XvP\_RLvP}} = -2/3\nu_{\text{GviP\_RLvP}} + 2/3\nu_{\text{HIS}_{\text{out}}} + 1/3\nu_{\text{PHE}_{\text{out}}} + 2/3\nu_{\text{RvP}_{\text{out}}} + \nu_{\text{TRP}_{\text{out}}} + 1/3\nu_{\text{TYR}_{\text{out}}}$

Transport relations:

$\nu_{\text{CO}_2\text{,out}} = -3\nu_{\text{ALA}_{\text{out}}} - 6\nu_{\text{ARG}_{\text{out}}} - 4\nu_{\text{ASN}_{\text{out}}} - 4\nu_{\text{ASP}_{\text{out}}} - 2\nu_{\text{AcCoA}_{\text{out}}} - 6\nu_{\text{FviP}_{\text{out}}} - 5\nu_{\text{GLN}_{\text{out}}} - 5\nu_{\text{GLU}_{\text{out}}} - 2\nu_{\text{GLY}_{\text{out}}} + 6\nu_{\text{GL}_{\text{in}}} - 3\nu_{\text{GiiiP}_{\text{out}}} - 6\nu_{\text{GviP}_{\text{out}}} - 6\nu_{\text{HIS}_{\text{out}}} - 6\nu_{\text{ILE}_{\text{out}}} - 6\nu_{\text{LEU}_{\text{out}}} - 6\nu_{\text{LYS}_{\text{out}}} - 5\nu_{\text{MET}_{\text{out}}} - 4\nu_{\text{OA}_{\text{out}}} - 9\nu_{\text{PHE}_{\text{out}}} - 5\nu_{\text{PRO}_{\text{out}}} - 5\nu_{\text{RvP}_{\text{out}}} - 3\nu_{\text{SER}_{\text{out}}} - 4\nu_{\text{THR}_{\text{out}}} - 11\nu_{\text{TRP}_{\text{out}}} - 9\nu_{\text{TYR}_{\text{out}}} - 5\nu_{\text{VAL}_{\text{out}}}$

Cytosolic-mitochondrial transport relations:

$\nu_{\text{AAo\_AAm}} = -\nu_{\text{AcCoA}_{\text{out}}} + \nu_{\text{B\_PY\_AcCoA\_LEU\_M}} + \nu_{\text{B\_THR\_GLY}} + \nu_{\text{IOSm\_IOSo}} - \nu_{\text{LEU}_{\text{out}}} - \nu_{\text{LYS}_{\text{out}}} + \nu_{\text{PYo\_AAo}}$

$\nu_{\text{ALAm\_ALAo}} = \nu_{\text{B\_PY\_ALA\_M1}}$

$\nu_{\text{CDm\_CDo}} = -3\nu_{\text{ALA}_{\text{out}}} - 4\nu_{\text{ARG}_{\text{out}}} - 3\nu_{\text{ASN}_{\text{out}}} - 3\nu_{\text{ASP}_{\text{out}}} - 2\nu_{\text{AcCoA}_{\text{out}}} + \nu_{\text{B\_PY\_AcCoA\_LEU\_M}} + 2\nu_{\text{B\_THR\_GLY}} - 6\nu_{\text{FviP}_{\text{out}}} - 4\nu_{\text{GLN}_{\text{out}}} - 4\nu_{\text{GLU}_{\text{out}}} - 3\nu_{\text{GLY}_{\text{out}}} + 6\nu_{\text{GL}_{\text{in}}} - 3\nu_{\text{GiiiP}_{\text{out}}} - \nu_{\text{GviP\_RLvP}} - 6\nu_{\text{GviP}_{\text{out}}} - 5\nu_{\text{HIS}_{\text{out}}} - 5\nu_{\text{ILE}_{\text{out}}} - 7\nu_{\text{LEU}_{\text{out}}} - 6\nu_{\text{LYS}_{\text{out}}} - 3\nu_{\text{MET}_{\text{out}}} - 3\nu_{\text{OA}_{\text{out}}} - 10\nu_{\text{PHE}_{\text{out}}} - 4\nu_{\text{PRO}_{\text{out}}} - \nu_{\text{PYo\_AAo}} - 5\nu_{\text{RvP}_{\text{out}}} - 3\nu_{\text{SER}_{\text{out}}} + \nu_{\text{TCA\_MAm\_PYm}} - 3\nu_{\text{THR}_{\text{out}}} - 12\nu_{\text{TRP}_{\text{out}}} - 10\nu_{\text{TYR}_{\text{out}}} - 5\nu_{\text{VAL}_{\text{out}}}$

$\nu_{\text{FUo\_FUm}} = \nu_{\text{US\_C\_ASP\_AS}}$

$\nu_{\text{GLNo\_GLNm}} = \nu_{\text{B\_GLT}}$

$\nu_{\text{GLUm\_GLUo}} = \nu_{\text{B\_GLT}} + \nu_{\text{GLN}_{\text{out}}} + \nu_{\text{GLU}_{\text{out}}} + \nu_{\text{LYS}_{\text{out}}} + \nu_{\text{OGo\_OGm}} + \nu_{\text{PRO}_{\text{out}}}$

$\nu_{\text{ILEm\_ILEo}} = \nu_{\text{ILE}_{\text{out}}}$

$\nu_{\text{LEUm\_LEUo}} = \nu_{\text{B\_PY\_AcCoA\_LEU\_M}}$

$\nu_{\text{MOm\_MOo}} = -\nu_{\text{B\_PY\_AcCoA\_LEU\_M}} - \nu_{\text{B\_PY\_VAL\_M}} - \nu_{\text{IOSm\_IOSo}} + \nu_{\text{LEU}_{\text{out}}} + \nu_{\text{VAL}_{\text{out}}}$

$\nu_{\text{OAo\_OAm}} = \nu_{\text{ARG}_{\text{out}}} + \nu_{\text{ASPm\_ASPo}} + \nu_{\text{GLN}_{\text{out}}} + \nu_{\text{GLU}_{\text{out}}} + \nu_{\text{LYS}_{\text{out}}} + \nu_{\text{PRO}_{\text{out}}} + \nu_{\text{TCA\_MAm\_PYm}} - \nu_{\text{US\_C\_ASP\_AS}}$

$\nu_{\text{ORm\_ORo}} = \nu_{\text{ARG}_{\text{out}}}$

$\nu_{\text{PYo\_PYm}} = -\nu_{\text{ALA}_{\text{out}}} - \nu_{\text{ARG}_{\text{out}}} - \nu_{\text{ASN}_{\text{out}}} - \nu_{\text{ASP}_{\text{out}}} + \nu_{\text{B\_PY\_ALA\_M1}} - 2\nu_{\text{FviP}_{\text{out}}} - \nu_{\text{GLN}_{\text{out}}} -$

$\nu_{\mathrm{GLU_{out}}} - \nu_{\mathrm{GLY_{out}}} + 2\nu_{\mathrm{GL_{in}}} - \nu_{\mathrm{GiiiP_{out}}} - 1/3\nu_{\mathrm{GviP\_RLvP}} - 2\nu_{\mathrm{GviP_{out}}} - 5/3\nu_{\mathrm{HIS_{out}}} - \nu_{\mathrm{ILE_{out}}} - \nu_{\mathrm{LYS_{out}}} - \nu_{\mathrm{MET_{out}}} - \nu_{\mathrm{OA_{out}}} - 10/3\nu_{\mathrm{PHE_{out}}} - \nu_{\mathrm{PRO_{out}}} - \nu_{\mathrm{PYo\_AAo}} - 5/3\nu_{\mathrm{RvP_{out}}} - \nu_{\mathrm{SER_{out}}} - \nu_{\mathrm{TCA\_MAm\_PYm}} - \nu_{\mathrm{THR_{out}}} - 4\nu_{\mathrm{TRP_{out}}} - 10/3\nu_{\mathrm{TYR_{out}}}$

$\nu_{\mathrm{THRo\_THRm}} = \nu_{\mathrm{ILE_{out}}}$

$\nu_{\mathrm{VALm\_VALo}} = \nu_{\mathrm{B\_PY\_VAL\_M}}$

Mitochondrial flux relations:

$\nu_{\mathrm{AAm\_ACm}} = -\nu_{\mathrm{AcCoA_{out}}} + \nu_{\mathrm{B\_PY\_AcCoA\_LEU\_M}} + \nu_{\mathrm{B\_THR\_GLY}} + \nu_{\mathrm{IOSm\_IOSo}} - \nu_{\mathrm{LEU_{out}}} - \nu_{\mathrm{LYS_{out}}} + \nu_{\mathrm{PYo\_AAo}}$

$\nu_{\mathrm{ACm\_AcCoAm}} = -\nu_{\mathrm{AcCoA_{out}}} + \nu_{\mathrm{B\_PY\_AcCoA\_LEU\_M}} + \nu_{\mathrm{B\_THR\_GLY}} + \nu_{\mathrm{IOSm\_IOSo}} - \nu_{\mathrm{LEU_{out}}} - \nu_{\mathrm{LYS_{out}}} + \nu_{\mathrm{PYo\_AAo}}$

$\nu_{\mathrm{B\_GLU\_OR}} = \nu_{\mathrm{ARG_{out}}}$

$\nu_{\mathrm{B\_OAm\_ASP\_01}} = \nu_{\mathrm{ASPm\_ASPo}}$

$\nu_{\mathrm{B\_OAm\_ASP\_02}} = \nu_{\mathrm{ASPm\_ASPo}}$

$\nu_{\mathrm{B\_OG\_GLU\_M}} = \nu_{\mathrm{ARG_{out}}} + \nu_{\mathrm{ASPm\_ASPo}} - \nu_{\mathrm{B\_GLT}} + \nu_{\mathrm{B\_PY\_ALA\_M1}} + \nu_{\mathrm{GLN_{out}}} + \nu_{\mathrm{GLU_{out}}} + \nu_{\mathrm{LYS_{out}}} + \nu_{\mathrm{OGo\_OGm}} + \nu_{\mathrm{PRO_{out}}}$

$\nu_{\mathrm{B\_PY\_ALA\_M2}} = \nu_{\mathrm{B\_PY\_ALA\_M1}}$

$\nu_{\mathrm{B\_PY\_AcCoA\_M}} = \nu_{\mathrm{B\_PY\_AcCoA\_LEU\_M}} + \nu_{\mathrm{IOSm\_IOSo}}$

$\nu_{\mathrm{B\_PY\_M1}} = \nu_{\mathrm{ILE_{out}}} + \nu_{\mathrm{LEU_{out}}} + \nu_{\mathrm{VAL_{out}}}$

$\nu_{\mathrm{B\_PY\_M2}} = \nu_{\mathrm{LEU_{out}}} + \nu_{\mathrm{VAL_{out}}}$

$\nu_{\mathrm{B\_PY\_THR\_ILE}} = \nu_{\mathrm{ILE_{out}}}$

$\nu_{\mathrm{GLUm\_OGm\_01}} = \nu_{\mathrm{ASPm\_ASPo}} + \nu_{\mathrm{B\_PY\_ALA\_M1}}$

$\nu_{\mathrm{GLUm\_OGm\_02}} = \nu_{\mathrm{ASPm\_ASPo}} + \nu_{\mathrm{B\_PY\_ALA\_M1}}$

$\nu_{\mathrm{TCA\_CIm\_OGm}} = -\nu_{\mathrm{ALA_{out}}} - \nu_{\mathrm{ARG_{out}}} - \nu_{\mathrm{ASN_{out}}} - \nu_{\mathrm{ASP_{out}}} - \nu_{\mathrm{AcCoA_{out}}} + \nu_{\mathrm{B\_THR\_GLY}} - 2\nu_{\mathrm{FviP_{out}}} - \nu_{\mathrm{GLN_{out}}} - \nu_{\mathrm{GLU_{out}}} - \nu_{\mathrm{GLY_{out}}} + 2\nu_{\mathrm{GL_{in}}} - \nu_{\mathrm{GiiiP_{out}}} - 1/3\nu_{\mathrm{GviP\_RLvP}} - 2\nu_{\mathrm{GviP_{out}}} - 5/3\nu_{\mathrm{HIS_{out}}} - 2\nu_{\mathrm{ILE_{out}}} - 3\nu_{\mathrm{LEU_{out}}} - 2\nu_{\mathrm{LYS_{out}}} - \nu_{\mathrm{MET_{out}}} - \nu_{\mathrm{OA_{out}}} - 10/3\nu_{\mathrm{PHE_{out}}} - \nu_{\mathrm{PRO_{out}}} - 5/3\nu_{\mathrm{RvP_{out}}} - \nu_{\mathrm{SER_{out}}} - \nu_{\mathrm{THR_{out}}} - 4\nu_{\mathrm{TRP_{out}}} - 10/3\nu_{\mathrm{TYR_{out}}} - 2\nu_{\mathrm{VAL_{out}}}$

$\nu_{\mathrm{TCA\_FUm\_MAm}} = -\nu_{\mathrm{ALA_{out}}} - 2\nu_{\mathrm{ARG_{out}}} - \nu_{\mathrm{ASN_{out}}} - \nu_{\mathrm{ASP_{out}}} - \nu_{\mathrm{AcCoA_{out}}} + \nu_{\mathrm{B\_THR\_GLY}} - 2\nu_{\mathrm{FviP_{out}}} - 2\nu_{\mathrm{GLN_{out}}} - 2\nu_{\mathrm{GLU_{out}}} - \nu_{\mathrm{GLY_{out}}} + 2\nu_{\mathrm{GL_{in}}} - \nu_{\mathrm{GiiiP_{out}}} - 1/3\nu_{\mathrm{GviP\_RLvP}} - 2\nu_{\mathrm{GviP_{out}}} - 5/3\nu_{\mathrm{HIS_{out}}} - 2\nu_{\mathrm{ILE_{out}}} - 3\nu_{\mathrm{LEU_{out}}} - 3\nu_{\mathrm{LYS_{out}}} - \nu_{\mathrm{MET_{out}}} - \nu_{\mathrm{OA_{out}}} - 10/3\nu_{\mathrm{PHE_{out}}} - 2\nu_{\mathrm{PRO_{out}}} - 5/3\nu_{\mathrm{RvP_{out}}} - \nu_{\mathrm{SER_{out}}} - \nu_{\mathrm{THR_{out}}} - 4\nu_{\mathrm{TRP_{out}}} - 10/3\nu_{\mathrm{TYR_{out}}} + \nu_{\mathrm{US\_C\_ASP\_AS}} - 2\nu_{\mathrm{VAL_{out}}}$

$\nu_{\mathrm{TCA\_MAm\_OAm}} = -\nu_{\mathrm{ALA_{out}}} - 2\nu_{\mathrm{ARG_{out}}} - \nu_{\mathrm{ASN_{out}}} - \nu_{\mathrm{ASP_{out}}} - \nu_{\mathrm{AcCoA_{out}}} + \nu_{\mathrm{B\_THR\_GLY}} - 2\nu_{\mathrm{FviP_{out}}} - 2\nu_{\mathrm{GLN_{out}}} - 2\nu_{\mathrm{GLU_{out}}} - \nu_{\mathrm{GLY_{out}}} + 2\nu_{\mathrm{GL_{in}}} - \nu_{\mathrm{GiiiP_{out}}} - 1/3\nu_{\mathrm{GviP\_RLvP}} - 2\nu_{\mathrm{GviP_{out}}} - 5/3\nu_{\mathrm{HIS_{out}}} - 2\nu_{\mathrm{ILE_{out}}} - 3\nu_{\mathrm{LEU_{out}}} - 3\nu_{\mathrm{LYS_{out}}} - \nu_{\mathrm{MET_{out}}} - \nu_{\mathrm{OA_{out}}} - 10/3\nu_{\mathrm{PHE_{out}}} - 2\nu_{\mathrm{PRO_{out}}} -$

$5/3\nu_{\mathrm{RvP_{out}}} - \nu_{\mathrm{SER_{out}}} - \nu_{\mathrm{TCA\_MAm\_PYm}} - \nu_{\mathrm{THR_{out}}} - 4\nu_{\mathrm{TRP_{out}}} - 10/3\nu_{\mathrm{TYR_{out}}} + \nu_{\mathrm{US\_C\_ASP\_AS}} - 2\nu_{\mathrm{VAL_{out}}}$

$\nu_{\mathrm{TCA\_OAm\_AcCoAm\_CIm}} = -\nu_{\mathrm{ALA_{out}}} - \nu_{\mathrm{ARG_{out}}} - \nu_{\mathrm{ASN_{out}}} - \nu_{\mathrm{ASP_{out}}} - \nu_{\mathrm{AcCoA_{out}}} + \nu_{\mathrm{B\_THR\_GLY}} - 2\nu_{\mathrm{FviP_{out}}} - \nu_{\mathrm{GLN_{out}}} - \nu_{\mathrm{GLU_{out}}} - \nu_{\mathrm{GLY_{out}}} + 2\nu_{\mathrm{GL_{in}}} - \nu_{\mathrm{GiiiP_{out}}} - 1/3\nu_{\mathrm{GviP\_RLvP}} - 2\nu_{\mathrm{GviP_{out}}} - 5/3\nu_{\mathrm{HIS_{out}}} - 2\nu_{\mathrm{ILE_{out}}} - 3\nu_{\mathrm{LEU_{out}}} - 2\nu_{\mathrm{LYS_{out}}} - \nu_{\mathrm{MET_{out}}} - \nu_{\mathrm{OA_{out}}} - 10/3\nu_{\mathrm{PHE_{out}}} - \nu_{\mathrm{PRO_{out}}} - 5/3\nu_{\mathrm{RvP_{out}}} - \nu_{\mathrm{SER_{out}}} - \nu_{\mathrm{THR_{out}}} - 4\nu_{\mathrm{TRP_{out}}} - 10/3\nu_{\mathrm{TYR_{out}}} - 2\nu_{\mathrm{VAL_{out}}}$

$\nu_{\mathrm{TCA\_OGm\_SUCoAm}} = -\nu_{\mathrm{ALA_{out}}} - 2\nu_{\mathrm{ARG_{out}}} - \nu_{\mathrm{ASN_{out}}} - \nu_{\mathrm{ASP_{out}}} - \nu_{\mathrm{AcCoA_{out}}} + \nu_{\mathrm{B\_THR\_GLY}} - 2\nu_{\mathrm{FviP_{out}}} - 2\nu_{\mathrm{GLN_{out}}} - 2\nu_{\mathrm{GLU_{out}}} - \nu_{\mathrm{GLY_{out}}} + 2\nu_{\mathrm{GL_{in}}} - \nu_{\mathrm{GiiiP_{out}}} - 1/3\nu_{\mathrm{GviP\_RLvP}} - 2\nu_{\mathrm{GviP_{out}}} - 5/3\nu_{\mathrm{HIS_{out}}} - 2\nu_{\mathrm{ILE_{out}}} - 3\nu_{\mathrm{LEU_{out}}} - 3\nu_{\mathrm{LYS_{out}}} - \nu_{\mathrm{MET_{out}}} - \nu_{\mathrm{OA_{out}}} - 10/3\nu_{\mathrm{PHE_{out}}} - 2\nu_{\mathrm{PRO_{out}}} - 5/3\nu_{\mathrm{RvP_{out}}} - \nu_{\mathrm{SER_{out}}} - \nu_{\mathrm{THR_{out}}} - 4\nu_{\mathrm{TRP_{out}}} - 10/3\nu_{\mathrm{TYR_{out}}} - 2\nu_{\mathrm{VAL_{out}}}$

$\nu_{\mathrm{TCA\_PYm\_AcCoAm}} = -\nu_{\mathrm{ALA_{out}}} - \nu_{\mathrm{ARG_{out}}} - \nu_{\mathrm{ASN_{out}}} - \nu_{\mathrm{ASP_{out}}} - 2\nu_{\mathrm{FviP_{out}}} - \nu_{\mathrm{GLN_{out}}} - \nu_{\mathrm{GLU_{out}}} - \nu_{\mathrm{GLY_{out}}} + 2\nu_{\mathrm{GL_{in}}} - \nu_{\mathrm{GiiiP_{out}}} - 1/3\nu_{\mathrm{GviP\_RLvP}} - 2\nu_{\mathrm{GviP_{out}}} - 5/3\nu_{\mathrm{HIS_{out}}} - 2\nu_{\mathrm{ILE_{out}}} - 2\nu_{\mathrm{LEU_{out}}} - \nu_{\mathrm{LYS_{out}}} - \nu_{\mathrm{MET_{out}}} - \nu_{\mathrm{OA_{out}}} - 10/3\nu_{\mathrm{PHE_{out}}} - \nu_{\mathrm{PRO_{out}}} - \nu_{\mathrm{PYo\_AAo}} - 5/3\nu_{\mathrm{RvP_{out}}} - \nu_{\mathrm{SER_{out}}} - \nu_{\mathrm{THR_{out}}} - 4\nu_{\mathrm{TRP_{out}}} - 10/3\nu_{\mathrm{TYR_{out}}} - 2\nu_{\mathrm{VAL_{out}}}$

$\nu_{\mathrm{TCA\_SUCoAm\_SUm}} = -\nu_{\mathrm{ALA_{out}}} - 2\nu_{\mathrm{ARG_{out}}} - \nu_{\mathrm{ASN_{out}}} - \nu_{\mathrm{ASP_{out}}} - \nu_{\mathrm{AcCoA_{out}}} + \nu_{\mathrm{B\_THR\_GLY}} - 2\nu_{\mathrm{FviP_{out}}} - 2\nu_{\mathrm{GLN_{out}}} - 2\nu_{\mathrm{GLU_{out}}} - \nu_{\mathrm{GLY_{out}}} + 2\nu_{\mathrm{GL_{in}}} - \nu_{\mathrm{GiiiP_{out}}} - 1/3\nu_{\mathrm{GviP\_RLvP}} - 2\nu_{\mathrm{GviP_{out}}} - 5/3\nu_{\mathrm{HIS_{out}}} - 2\nu_{\mathrm{ILE_{out}}} - 3\nu_{\mathrm{LEU_{out}}} - 3\nu_{\mathrm{LYS_{out}}} - \nu_{\mathrm{MET_{out}}} - \nu_{\mathrm{OA_{out}}} - 10/3\nu_{\mathrm{PHE_{out}}} - 2\nu_{\mathrm{PRO_{out}}} - 5/3\nu_{\mathrm{RvP_{out}}} - \nu_{\mathrm{SER_{out}}} - \nu_{\mathrm{THR_{out}}} - 4\nu_{\mathrm{TRP_{out}}} - 10/3\nu_{\mathrm{TYR_{out}}} - 2\nu_{\mathrm{VAL_{out}}}$

$\nu_{\mathrm{TCA\_SUm\_FUm}} = -\nu_{\mathrm{ALA_{out}}} - 2\nu_{\mathrm{ARG_{out}}} - \nu_{\mathrm{ASN_{out}}} - \nu_{\mathrm{ASP_{out}}} - \nu_{\mathrm{AcCoA_{out}}} + \nu_{\mathrm{B\_THR\_GLY}} - 2\nu_{\mathrm{FviP_{out}}} - 2\nu_{\mathrm{GLN_{out}}} - 2\nu_{\mathrm{GLU_{out}}} - \nu_{\mathrm{GLY_{out}}} + 2\nu_{\mathrm{GL_{in}}} - \nu_{\mathrm{GiiiP_{out}}} - 1/3\nu_{\mathrm{GviP\_RLvP}} - 2\nu_{\mathrm{GviP_{out}}} - 5/3\nu_{\mathrm{HIS_{out}}} - 2\nu_{\mathrm{ILE_{out}}} - 3\nu_{\mathrm{LEU_{out}}} - 3\nu_{\mathrm{LYS_{out}}} - \nu_{\mathrm{MET_{out}}} - \nu_{\mathrm{OA_{out}}} - 10/3\nu_{\mathrm{PHE_{out}}} - 2\nu_{\mathrm{PRO_{out}}} - 5/3\nu_{\mathrm{RvP_{out}}} - \nu_{\mathrm{SER_{out}}} - \nu_{\mathrm{THR_{out}}} - 4\nu_{\mathrm{TRP_{out}}} - 10/3\nu_{\mathrm{TYR_{out}}} - 2\nu_{\mathrm{VAL_{out}}}$

3.2 Constraints

The following constraints are implications of irreversibility conditions (positiveness of unidirectional fluxes) in the system of reactions:

$3\nu_{\mathrm{ALA_{out}}} + 3\nu_{\mathrm{ARG_{out}}} + 3\nu_{\mathrm{ASN_{out}}} + 3\nu_{\mathrm{ASP_{out}}} + 6\nu_{\mathrm{FviP_{out}}} + 3\nu_{\mathrm{GLN_{out}}} + 3\nu_{\mathrm{GLU_{out}}} + 3\nu_{\mathrm{GLY_{out}}} + 3\nu_{\mathrm{GiiiP_{out}}} + \nu_{\mathrm{GviP\_RLvP}} + 6\nu_{\mathrm{GviP_{out}}} + 5\nu_{\mathrm{HIS_{out}}} + 6\nu_{\mathrm{ILE_{out}}} + 6\nu_{\mathrm{LEU_{out}}} + 3\nu_{\mathrm{LYS_{out}}} + 3\nu_{\mathrm{MET_{out}}} + 3\nu_{\mathrm{OA_{out}}} + 10\nu_{\mathrm{PHE_{out}}} + 3\nu_{\mathrm{PRO_{out}}} + 3\nu_{\mathrm{PYo\_AAo}} + 5\nu_{\mathrm{RvP_{out}}} + 3\nu_{\mathrm{SER_{out}}} + 3\nu_{\mathrm{THR_{out}}} + 12\nu_{\mathrm{TRP_{out}}} + 10\nu_{\mathrm{TYR_{out}}} + 6\nu_{\mathrm{VAL_{out}}} \leq 6\nu_{\mathrm{GL_{in}}}$

$3\nu_{\mathrm{ALA_{out}}} + 6\nu_{\mathrm{ARG_{out}}} + 3\nu_{\mathrm{ASN_{out}}} + 3\nu_{\mathrm{ASP_{out}}} + 3\nu_{\mathrm{AcCoA_{out}}} + 6\nu_{\mathrm{FviP_{out}}} + 6\nu_{\mathrm{GLN_{out}}} + 6\nu_{\mathrm{GLU_{out}}} + 3\nu_{\mathrm{GLY_{out}}} + 3\nu_{\mathrm{GiiiP_{out}}} + \nu_{\mathrm{GviP\_RLvP}} + 6\nu_{\mathrm{GviP_{out}}} + 5\nu_{\mathrm{HIS_{out}}} + 6\nu_{\mathrm{ILE_{out}}} + 9\nu_{\mathrm{LEU_{out}}} + 9\nu_{\mathrm{LYS_{out}}} + 3\nu_{\mathrm{MET_{out}}} + 3\nu_{\mathrm{OA_{out}}} + 10\nu_{\mathrm{PHE_{out}}} + 6\nu_{\mathrm{PRO_{out}}} + 5\nu_{\mathrm{RvP_{out}}} + 3\nu_{\mathrm{SER_{out}}} + 3\nu_{\mathrm{THR_{out}}} + 12\nu_{\mathrm{TRP_{out}}} + 10\nu_{\mathrm{TYR_{out}}} + 6\nu_{\mathrm{VAL_{out}}} \leq 3\nu_{\mathrm{B\_THR\_GLY}} + 6\nu_{\mathrm{GL_{in}}}$

$3/2\nu_{\mathrm{ALA_{out}}} + 3\nu_{\mathrm{ARG_{out}}} + 2\nu_{\mathrm{ASN_{out}}} + 2\nu_{\mathrm{ASP_{out}}} + \nu_{\mathrm{AcCoA_{out}}} + 3\nu_{\mathrm{FviP_{out}}} + 5/2\nu_{\mathrm{GLN_{out}}} + 5/2\nu_{\mathrm{GLU_{out}}} + \nu_{\mathrm{GLY_{out}}} + 3/2\nu_{\mathrm{GiiiP_{out}}} + 3\nu_{\mathrm{GviP_{out}}} + 3\nu_{\mathrm{HIS_{out}}} + 3\nu_{\mathrm{ILE_{out}}} + 3\nu_{\mathrm{LEU_{out}}} + 3\nu_{\mathrm{LYS_{out}}} + 5/2\nu_{\mathrm{MET_{out}}} + 2\nu_{\mathrm{OA_{out}}} + 9/2\nu_{\mathrm{PHE_{out}}} + 5/2\nu_{\mathrm{PRO_{out}}} + 5/2\nu_{\mathrm{RvP_{out}}} + 3/2\nu_{\mathrm{SER_{out}}} + 2\nu_{\mathrm{THR_{out}}} + 11/2\nu_{\mathrm{TRP_{out}}} + 9/2\nu_{\mathrm{TYR_{out}}} + 5/2\nu_{\mathrm{VAL_{out}}} \leq 3\nu_{\mathrm{GL_{in}}}$

$\nu_{\mathrm{ARG_{out}}} \leq \nu_{\mathrm{US\_C\_ASP\_AS}}$

$\nu_{\mathrm{B\_PY\_AcCoA\_LEU\_M}} \leq \nu_{\mathrm{LEU_{out}}}$

$\nu_{\mathrm{B\_THR\_GLY}} \leq \nu_{\mathrm{GLY_{out}}}$

4 Values of independent fluxes used to generate Figure 1.

External flux	Value (a.u.)	Internal flux	Value (a.u.)
$\nu_{\mathrm{ALA_{out}}}$	2.77	$\nu_{\mathrm{ASPm\_ASPo}}$	2
$\nu_{\mathrm{ARG_{out}}}$	1.94	$\nu_{\mathrm{B\_GLT}}$	4
$\nu_{\mathrm{ASN_{out}}}$	0.82	$\nu_{\mathrm{B\_PY\_ALA\_M1}}$	1.0
$\nu_{\mathrm{ASP_{out}}}$	2.39	$\nu_{\mathrm{B\_PY\_AcCoA\_LEU\_M}}$	2
$\nu_{\mathrm{AcCoA_{out}}}$	0.3	$\nu_{\mathrm{B\_PY\_VAL\_M}}$	1
$\nu_{\mathrm{FviP_{out}}}$	11	$\nu_{\mathrm{B\_THR\_GLY}}$	0
$\nu_{\mathrm{GLN_{out}}}$	1.06	$\nu_{\mathrm{GviP\_RLvP}}$	5
$\nu_{\mathrm{GLU_{out}}}$	3.04	$\nu_{\mathrm{IOSm\_IOSo}}$	0
$\nu_{\mathrm{GLY_{out}}}$	1.17	$\nu_{\mathrm{OA\_PEP}}$	0
$\nu_{\mathrm{GL_{in}}}$	100	$\nu_{\mathrm{OGo\_OGm}}$	0
$\nu_{\mathrm{GiiiP_{out}}}$	0.45	$\nu_{\mathrm{PYo\_AAo}}$	10
$\nu_{\mathrm{GviP_{out}}}$	3.8	$\nu_{\mathrm{TCA\_MAm\_PYm}}$	0
$\nu_{\mathrm{HIS_{out}}}$	0.8	$\nu_{\mathrm{US\_C\_ASP\_AS}}$	2.5
$\nu_{\mathrm{ILE_{out}}}$	2.33		
$\nu_{\mathrm{LEU_{out}}}$	3.57		
$\nu_{\mathrm{LYS_{out}}}$	3.45		
$\nu_{\mathrm{MET_{out}}}$	0.51		
$\nu_{\mathrm{OA_{out}}}$	0.36		
$\nu_{\mathrm{PHE_{out}}}$	2.43		
$\nu_{\mathrm{PRO_{out}}}$	1.66		
$\nu_{\mathrm{RvP_{out}}}$	2.6		
$\nu_{\mathrm{SER_{out}}}$	1.12		
$\nu_{\mathrm{THR_{out}}}$	1.54		
$\nu_{\mathrm{TRP_{out}}}$	0.62		
$\nu_{\mathrm{TYR_{out}}}$	1.84		
$\nu_{\mathrm{VAL_{out}}}$	2.66		

5 Symbolic solution of the steady state problem with measured values

5.1 Flux relations

Final flux values after substituting all specified independent flux values from the above Table are shown in bold.

Cytosolic flux relations:

$\nu_{\text{AAo\_ACo}} = 7.320 - \nu_{\text{B\_PY\_AcCoA\_LEU\_M}} - \nu_{\text{B\_THR\_GLY}} - \nu_{\text{IOSm\_IOSo}} = \mathbf{5.320}$

$\nu_{\text{ACo\_AcCoAo}} = 7.830 - \nu_{\text{B\_PY\_AcCoA\_LEU\_M}} - \nu_{\text{IOSm\_IOSo}} = \mathbf{5.830}$

$\nu_{\text{BCo\_CPo\_01}} = \nu_{\text{US\_C\_ASP\_AS}} = \mathbf{2.500}$

$\nu_{\text{BCo\_CPo\_02}} = \nu_{\text{US\_C\_ASP\_AS}} = \mathbf{2.500}$

$\nu_{\text{B\_AC\_01}} = 0.510 = \mathbf{0.510}$

$\nu_{\text{B\_ASP\_ASN}} = 0.820 = \mathbf{0.820}$

$\nu_{\text{B\_ASP\_THR}} = 3.870 + \nu_{\text{B\_THR\_GLY}} = \mathbf{3.870}$

$\nu_{\text{B\_AcCoA\_01}} = 0.510 = \mathbf{0.510}$

$\nu_{\text{B\_GLU\_GLN}} = 1.060 + \nu_{\text{B\_GLT}} + \nu_{\text{US\_C\_ASP\_AS}} = \mathbf{7.560}$

$\nu_{\text{B\_GLU\_PRO}} = 1.660 = \mathbf{1.660}$

$\nu_{\text{B\_GiiiP\_GLY}} = 1.170 - \nu_{\text{B\_THR\_GLY}} = \mathbf{1.170}$

$\nu_{\text{B\_GiiiP\_SER}} = 2.910 - \nu_{\text{B\_THR\_GLY}} = \mathbf{2.910}$

$\nu_{\text{B\_LYS\_C1}} = 3.450 = \mathbf{3.450}$

$\nu_{\text{B\_LYS\_C2}} = 3.450 = \mathbf{3.450}$

$\nu_{\text{B\_OA\_AcCoA\_MET}} = 0.510 = \mathbf{0.510}$

$\nu_{\text{B\_OAo\_ASP\_01}} = 7.590 - \nu_{\text{ASPm\_ASPo}} + \nu_{\text{B\_THR\_GLY}} + \nu_{\text{US\_C\_ASP\_AS}} = \mathbf{8.090}$

$\nu_{\text{B\_OAo\_ASP\_02}} = 7.590 - \nu_{\text{ASPm\_ASPo}} + \nu_{\text{B\_THR\_GLY}} + \nu_{\text{US\_C\_ASP\_AS}} = \mathbf{8.090}$

$\nu_{\text{B\_OG\_GLU\_C}} = 6.910 - \nu_{\text{ASPm\_ASPo}} - \nu_{\text{B\_PY\_ALA\_M1}} + \nu_{\text{B\_THR\_GLY}} - \nu_{\text{OGo\_OGm}} + \nu_{\text{US\_C\_ASP\_AS}} = \mathbf{6.410}$

$\nu_{\text{B\_PEP\_01}} = 4.890 = \mathbf{4.890}$

$\nu_{\text{B\_PEP\_EivP\_01}} = 4.890 = \mathbf{4.890}$

$\nu_{\text{B\_PEP\_EivP\_PHE}} = 2.430 = \mathbf{2.430}$

$\nu_{\text{B\_PEP\_EivP\_TRP\_01}} = 0.620 = \mathbf{0.620}$

$\nu_{\text{B\_PEP\_EivP\_TRP\_02}} = 0.620 = \mathbf{0.620}$

$\nu_{\text{B\_PEP\_EivP\_TRP\_03}} = 0.620 = \mathbf{0.620}$

$\nu_{\text{B\_PEP\_EivP\_TRP\_04}} = 0.620 = \mathbf{0.620}$

$\nu_{\text{B\_PEP\_EivP\_TRP\_05}} = 0.620 = \mathbf{0.620}$

$\nu_{\text{B\_PEP\_EivP\_TYR}} = 1.840 = \mathbf{1.840}$

$\nu_{\text{B\_PY\_ALA\_C1}} = 2.770 - \nu_{\text{B\_PY\_ALA\_M1}} = \mathbf{1.770}$

$\nu_{\text{B\_PY\_ALA\_C2}} = 2.770 - \nu_{\text{B\_PY\_ALA\_M1}} = \mathbf{1.770}$

$\nu_{\text{B\_PY\_AcCoA\_C}} = 3.570 - \nu_{\text{B\_PY\_AcCoA\_LEU\_M}} - \nu_{\text{IOSm\_IOSo}} = \mathbf{1.570}$

$\nu_{\text{B\_PY\_AcCoA\_LEU\_C}} = 3.570 - \nu_{\text{B\_PY\_AcCoA\_LEU\_M}} = \mathbf{1.570}$

$\nu_{\text{B\_PY\_VAL\_C}} = 2.660 - \nu_{\text{B\_PY\_VAL\_M}} = \mathbf{1.660}$

$\nu_{\text{B\_RvP\_HIS}} = 0.800 = \mathbf{0.800}$

$\nu_{\text{CD\_BC}} = 20.410 + \nu_{\text{B\_THR\_GLY}} + \nu_{\text{OA\_PEP}} + \nu_{\text{TCA\_MAm\_PYm}} + \nu_{\text{US\_C\_ASP\_AS}} = \mathbf{22.910}$

$\nu_{\text{FviP\_GP\_GiiiP}} = 80.890 - 1/3\nu_{\text{GviP\_RLvP}} = \mathbf{79.223}$

$\nu_{\text{GLUo\_OGo\_01}} = 10.360 - \nu_{\text{ASPm\_ASPo}} - \nu_{\text{B\_PY\_ALA\_M1}} + \nu_{\text{B\_THR\_GLY}} + \nu_{\text{US\_C\_ASP\_AS}} = \mathbf{9.860}$

$\nu_{\text{GLUo\_OGo\_02}} = 10.360 - \nu_{\text{ASPm\_ASPo}} - \nu_{\text{B\_PY\_ALA\_M1}} + \nu_{\text{B\_THR\_GLY}} + \nu_{\text{US\_C\_ASP\_AS}} = \mathbf{9.860}$

$\nu_{\text{GL\_GviP}} = 100.000 = \mathbf{100.000}$

$\nu_{\text{GP\_GiiiP}} = 80.890 - 1/3\nu_{\text{GviP\_RLvP}} = \mathbf{79.223}$

$\nu_{\text{GiiiP\_PEP}} = 154.440 + \nu_{\text{B\_THR\_GLY}} - 1/3\nu_{\text{GviP\_RLvP}} = \mathbf{152.773}$

$\nu_{\text{GviP\_FviP}} = 96.200 - \nu_{\text{GviP\_RLvP}} = \mathbf{91.200}$

$\nu_{\text{PEP\_PY}} = 144.660 + \nu_{\text{B\_THR\_GLY}} - 1/3\nu_{\text{GviP\_RLvP}} + \nu_{\text{OA\_PEP}} = \mathbf{142.993}$

$\nu_{\text{PPP\_FviP\_EivP\_SviiP\_GiiiP\_01}} = 0.290 + 1/3\nu_{\text{GviP\_RLvP}} = \mathbf{1.957}$

$\nu_{\text{PPP\_FviP\_EivP\_SviiP\_GiiiP\_02}} = 0.290 + 1/3\nu_{\text{GviP\_RLvP}} = \mathbf{1.957}$

$\nu_{\text{PPP\_FviP\_GiiiP\_XvP\_EivP\_01}} = 4.600 - 1/3\nu_{\text{GviP\_RLvP}} = \mathbf{2.933}$

$\nu_{\text{PPP\_FviP\_GiiiP\_XvP\_EivP\_02}} = 4.600 - 1/3\nu_{\text{GviP\_RLvP}} = \mathbf{2.933}$

$\nu_{\text{PPP\_SviiP\_GiiiP\_XvP\_RvP\_01}} = 0.290 + 1/3\nu_{\text{GviP\_RLvP}} = \mathbf{1.957}$

$\nu_{\text{PPP\_SviiP\_GiiiP\_XvP\_RvP\_02}} = 0.290 + 1/3\nu_{\text{GviP\_RLvP}} = \mathbf{1.957}$

$\nu_{\text{PYo\_OAo}} = 19.100 + \nu_{\text{B\_THR\_GLY}} + \nu_{\text{OA\_PEP}} + \nu_{\text{TCA\_MAm\_PYm}} = \mathbf{19.100}$

$\nu_{\text{RLvP\_RvP}} = 4.310 + 1/3\nu_{\text{GviP\_RLvP}} = \mathbf{5.977}$

$\nu_{\text{US\_ARG\_OR}} = -1.940 + \nu_{\text{US\_C\_ASP\_AS}} = \mathbf{0.560}$

$\nu_{\text{US\_AS\_FU\_ARG}} = \nu_{\text{US\_C\_ASP\_AS}} = \mathbf{2.500}$

$\nu_{\mathrm{US\_OR\_C}} = \nu_{\mathrm{US\_C\_ASP\_AS}} = \mathbf{2.500}$

$\nu_{\mathrm{XvP\_RLvP}} = 4.310 - 2/3\nu_{\mathrm{GviP\_RLvP}} = \mathbf{0.977}$

Transport relations:

$\nu_{\mathrm{CO_2,out}} = 299.360 = \mathbf{299.360}$

Cytosolic-mitochondrial transport relations:

$\nu_{\mathrm{AAo\_AAm}} = -7.320 + \nu_{\mathrm{B\_PY\_AcCoA\_LEU\_M}} + \nu_{\mathrm{B\_THR\_GLY}} + \nu_{\mathrm{IOSm\_IOSo}} + \nu_{\mathrm{PYo\_AAo}} = \mathbf{4.680}$

$\nu_{\mathrm{ALAm\_ALAo}} = \nu_{\mathrm{B\_PY\_ALA\_M1}} = \mathbf{1.000}$

$\nu_{\mathrm{CDm\_CDo}} = 308.630 + \nu_{\mathrm{B\_PY\_AcCoA\_LEU\_M}} + 2\nu_{\mathrm{B\_THR\_GLY}} - \nu_{\mathrm{GviP\_RLvP}} - \nu_{\mathrm{PYo\_AAo}} + \nu_{\mathrm{TCA\_MAm\_PYm}} = \mathbf{295.630}$

$\nu_{\mathrm{FUo\_FUm}} = \nu_{\mathrm{US\_C\_ASP\_AS}} = \mathbf{2.500}$

$\nu_{\mathrm{GLNo\_GLNm}} = \nu_{\mathrm{B\_GLT}} = \mathbf{4.000}$

$\nu_{\mathrm{GLUm\_GLUo}} = 9.210 + \nu_{\mathrm{B\_GLT}} + \nu_{\mathrm{OGo\_OGm}} = \mathbf{13.210}$

$\nu_{\mathrm{ILEm\_ILEo}} = 2.330 = \mathbf{2.330}$

$\nu_{\mathrm{LEUm\_LEUo}} = \nu_{\mathrm{B\_PY\_AcCoA\_LEU\_M}} = \mathbf{2.000}$

$\nu_{\mathrm{MOm\_MOo}} = 6.230 - \nu_{\mathrm{B\_PY\_AcCoA\_LEU\_M}} - \nu_{\mathrm{B\_PY\_VAL\_M}} - \nu_{\mathrm{IOSm\_IOSo}} = \mathbf{3.230}$

$\nu_{\mathrm{OAo\_OAm}} = 11.150 + \nu_{\mathrm{ASPm\_ASPo}} + \nu_{\mathrm{TCA\_MAm\_PYm}} - \nu_{\mathrm{US\_C\_ASP\_AS}} = \mathbf{10.650}$

$\nu_{\mathrm{ORm\_ORo}} = 1.940 = \mathbf{1.940}$

$\nu_{\mathrm{PYo\_PYm}} = 123.410 + \nu_{\mathrm{B\_PY\_ALA\_M1}} - 1/3\nu_{\mathrm{GviP\_RLvP}} - \nu_{\mathrm{PYo\_AAo}} - \nu_{\mathrm{TCA\_MAm\_PYm}} = \mathbf{112.743}$

$\nu_{\mathrm{THRo\_THRm}} = 2.330 = \mathbf{2.330}$

$\nu_{\mathrm{VALm\_VALo}} = \nu_{\mathrm{B\_PY\_VAL\_M}} = \mathbf{1.000}$

Mitochondrial flux relations:

$\nu_{\mathrm{AAm\_ACm}} = -7.320 + \nu_{\mathrm{B\_PY\_AcCoA\_LEU\_M}} + \nu_{\mathrm{B\_THR\_GLY}} + \nu_{\mathrm{IOSm\_IOSo}} + \nu_{\mathrm{PYo\_AAo}} = \mathbf{4.680}$

$\nu_{\mathrm{ACm\_AcCoAm}} = -7.320 + \nu_{\mathrm{B\_PY\_AcCoA\_LEU\_M}} + \nu_{\mathrm{B\_THR\_GLY}} + \nu_{\mathrm{IOSm\_IOSo}} + \nu_{\mathrm{PYo\_AAo}} = \mathbf{4.680}$

$\nu_{\mathrm{B\_GLU\_OR}} = 1.940 = \mathbf{1.940}$

$\nu_{\mathrm{B\_OAm\_ASP\_01}} = \nu_{\mathrm{ASPm\_ASPo}} = \mathbf{2.000}$

$\nu_{\mathrm{B\_OAm\_ASP\_02}} = \nu_{\mathrm{ASPm\_ASPo}} = \mathbf{2.000}$

$\nu_{\mathrm{B\_OG\_GLU\_M}} = 11.150 + \nu_{\mathrm{ASPm\_ASPo}} - \nu_{\mathrm{B\_GLT}} + \nu_{\mathrm{B\_PY\_ALA\_M1}} + \nu_{\mathrm{OGo\_OGm}} = \mathbf{10.150}$

$\nu_{\mathrm{B\_PY\_ALA\_M2}} = \nu_{\mathrm{B\_PY\_ALA\_M1}} = \mathbf{1.000}$

$\nu_{\mathrm{B\_PY\_AcCoA\_M}} = \nu_{\mathrm{B\_PY\_AcCoA\_LEU\_M}} + \nu_{\mathrm{IOSm\_IOSo}} = \mathbf{2.000}$

$\nu_{\text{B\_PY\_M1}} = 8.560 = \mathbf{8.560}$

$\nu_{\text{B\_PY\_M2}} = 6.230 = \mathbf{6.230}$

$\nu_{\text{B\_PY\_THR\_ILE}} = 2.330 = \mathbf{2.330}$

$\nu_{\text{GLUm\_OGm\_01}} = \nu_{\text{ASPm\_ASPo}} + \nu_{\text{B\_PY\_ALA\_M1}} = \mathbf{3.000}$

$\nu_{\text{GLUm\_OGm\_02}} = \nu_{\text{ASPm\_ASPo}} + \nu_{\text{B\_PY\_ALA\_M1}} = \mathbf{3.000}$

$\nu_{\text{TCA\_CIm\_OGm}} = 101.300 + \nu_{\text{B\_THR\_GLY}} - 1/3\nu_{\text{GviP\_RLvP}} = \mathbf{99.633}$

$\nu_{\text{TCA\_FUm\_MAm}} = 90.150 + \nu_{\text{B\_THR\_GLY}} - 1/3\nu_{\text{GviP\_RLvP}} + \nu_{\text{US\_C\_ASP\_AS}} = \mathbf{90.983}$

$\nu_{\text{TCA\_MAm\_OAm}} = 90.150 + \nu_{\text{B\_THR\_GLY}} - 1/3\nu_{\text{GviP\_RLvP}} - \nu_{\text{TCA\_MAm\_PYm}} + \nu_{\text{US\_C\_ASP\_AS}} = \mathbf{90.983}$

$\nu_{\text{TCA\_OAm\_AcCoAm\_CIm}} = 101.300 + \nu_{\text{B\_THR\_GLY}} - 1/3\nu_{\text{GviP\_RLvP}} = \mathbf{99.633}$

$\nu_{\text{TCA\_OGm\_SUCoAm}} = 90.150 + \nu_{\text{B\_THR\_GLY}} - 1/3\nu_{\text{GviP\_RLvP}} = \mathbf{88.483}$

$\nu_{\text{TCA\_PYm\_AcCoAm}} = 108.620 - 1/3\nu_{\text{GviP\_RLvP}} - \nu_{\text{PYo\_AAo}} = \mathbf{96.953}$

$\nu_{\text{TCA\_SUCoAm\_SUm}} = 90.150 + \nu_{\text{B\_THR\_GLY}} - 1/3\nu_{\text{GviP\_RLvP}} = \mathbf{88.483}$

$\nu_{\text{TCA\_SUm\_FUm}} = 90.150 + \nu_{\text{B\_THR\_GLY}} - 1/3\nu_{\text{GviP\_RLvP}} = \mathbf{88.483}$

5.2 Constraints

$1.94 \leq \nu_{\text{US\_C\_ASP\_AS}}$

$\nu_{\text{B\_PY\_AcCoA\_LEU\_M}} \leq 3.57$

$\nu_{\text{B\_THR\_GLY}} \leq 1.17$

$\nu_{\text{GviP\_RLvP}} + 3.0\nu_{\text{PYo\_AAo}} \leq 325.86$

$\nu_{\text{GviP\_RLvP}} \leq 270.45 + 3.0\nu_{\text{B\_THR\_GLY}}$

Table 3: Extreme independent fluxes

Fluxes	1	2	3	4	5	6	7	8	9	10	11	12
$\nu_{\mathrm{ASPm\_ASPo}}$	0.0	0.0	0.0	0.0	0.0	0.0	0.0	0.0	0.0	0.0	0.0	0.0
$\nu_{\mathrm{B\_GLT}}$	-70.15	21.17	21.17	-70.15	21.17	21.17	21.17	21.17	-70.15	21.17	21.17	-70.15
$\nu_{\mathrm{B\_PY\_ALA\_M1}}$	2.77	2.77	2.77	2.77	2.77	2.77	2.77	2.77	2.77	2.77	2.77	2.77
$\nu_{\mathrm{B\_PY\_AcCoA\_LEU\_M}}$	3.57	3.57	3.57	3.57	3.57	3.57	0.0	0.0	0.0	0.0	0.0	0.0
$\nu_{\mathrm{B\_PY\_VAL\_M}}$	2.66	2.66	2.66	2.66	2.66	2.66	2.66	2.66	2.66	2.66	2.66	2.66
$\nu_{\mathrm{B\_THR\_GLY}}$	-90.15	1.17	1.17	-90.15	1.17	1.17	1.17	1.17	-90.15	1.17	1.17	-90.15
$\nu_{\mathrm{GviP\_RLvP}}$	0.0	0.0	0.0	0.0	273.96	273.96	273.96	273.96	0.0	0.0	0.0	0.0
$\nu_{\mathrm{IOSm\_IOSo}}$	93.9	2.58	-106.04	-14.72	-14.72	2.58	-11.15	6.15	-11.15	-102.47	6.15	97.47
$\nu_{\mathrm{OA\_PEP}}$	0.0	0.0	0.0	0.0	0.0	0.0	0.0	0.0	0.0	0.0	0.0	0.0
$\nu_{\mathrm{OGo\_OGm}}$	-84.07	7.25	7.25	-84.07	7.25	7.25	7.25	7.25	-84.07	7.25	7.25	-84.07
$\nu_{\mathrm{PYo\_AAo}}$	0.0	0.0	108.62	108.62	17.3	0.0	17.3	0.0	108.62	108.62	0.0	0.0
$\nu_{\mathrm{TCA\_MAm\_PYm}}$	0.0	0.0	0.0	0.0	0.0	0.0	0.0	0.0	0.0	0.0	0.0	0.0
$\nu_{\mathrm{US\_C\_ASP\_AS}}$	1.94	1.94	1.94	1.94	1.94	1.94	1.94	1.94	1.94	1.94	1.94	1.94
